# pH-Responsive Nanostructured Calcium Phosphate Microrods as Pulmonary Delivery Platform: Fabrication, Characterization, and Comparative Assessment of Cytotoxic and Transcriptomic Responses in Alveolar Macrophages

**DOI:** 10.3390/pharmaceutics18040428

**Published:** 2026-03-31

**Authors:** Jannis Fries, Richard Bachmann, Amalia Schechtel, Oliver Janka, Julia Schulze-Hentrich, Marc Schneider

**Affiliations:** 1 Department of Pharmacy, Biopharmaceutics and Pharmaceutical Technology, Saarland University, Campus C4 1, 66123 Saarbrücken, Germany; jannis.fries@uni-saarland.de (J.F.); amalia.schechtel@uni-saarland.de (A.S.); 2 PharmaScienceHub (PSH), 66123 Saarbrücken, Germany; richard.bachmann@uni-saarland.de (R.B.); julia.schulze-hentrich@uni-saarland.de (J.S.-H.); 3 Department of Genetics and Epigenetics, Saarland University, Campus A2 4, 66123 Saarbrücken, Germany; 4 Department of Chemistry, Inorganic Solid-State Chemistry, Saarland University, Campus C4 1, 66123 Saarbrücken, Germany; oliver.janka@uni-saarland.de

**Keywords:** microcylinders, calcium phosphate, aerodynamic properties, smart delivery, cell interaction, RNA-seq, alveolar mouse macrophages, pH responsiveness

## Abstract

**Background:** Nanostructured, rod-shaped microparticles represent a promising drug delivery platform for the pulmonary delivery and targeting of alveolar macrophages by exploiting the aerodynamic advantages of fiber-like geometries. These microrods feature a hierarchical architecture, designed for potential macromolecular payloads, and silica (SiO_2_)-based systems have previously been shown to successfully deliver oligonucleotides in vitro. However, current microrod systems mainly rely on nanoparticulate SiO_2_-based frameworks with limited biodegradability and lack a specific escape mechanism to the cytosol. Therefore, a nanostructured calcium phosphate (CaP) framework is proposed as a biodegradable and resorbable alternative, featuring pH-responsive dissolution under endolysosomal conditions. **Methods and Results:** This study presents the fabrication of nanostructured, rod-shaped calcium phosphate microparticles and discusses their suitability as a potential pulmonary drug delivery platform. The particles feature dissolution-driven disintegration in acidic and ion-rich environments relevant to phagolysosomes. In addition, the particles exhibited a favorable acute cytotoxicity profile in the murine alveolar macrophage cell line MH-S compared with their SiO_2_-based counterparts. Comparative RNA-seq analysis of MH-S exposed to the particles indicates a mild transcriptomic response, while canonical signatures of classical or alternative macrophage activation programs were not observed, supporting a generally well-tolerated exposure profile of the carrier. **Conclusions:** Together, these findings establish key prerequisites for employing calcium phosphate microrods as a biodegradable pulmonary carrier platform in subsequent studies incorporating therapeutic cargos.

## 1. Introduction

The persistent high prevalence and mortality of lung diseases, ranging from chronic disorders to respiratory infections and malignancies, underline the need for advanced therapeutic approaches [1,2,3,4]. Pharmaceutical research has recently focused on targeted delivery of macromolecular drugs, a heterogeneous group of biotherapeutics, which includes nucleic acids, peptides and antibodies [5]. While these modalities offer promising strategies, their effective delivery remains challenging because of their physicochemical instability, enzymatic susceptibility, poor cell penetration capabilities, inefficient endosomal escape, and safety concerns related to many delivery systems [5,6,7].

In this context, the alveolar macrophage (AM) represents a promising therapeutic target because of its role in a variety of pulmonary diseases, including infections, inflammatory disorders and lung cancer [8,9,10]. AMs are tissue-resident immune cells that are mainly localized on the luminal side of the alveolar epithelium but also occur in the smaller conducting airways [11]. As gatekeepers of the lower respiratory tract, AMs have essential roles in clearing the lung of inhaled particles or microbes and modulating immune responses, thereby contributing to lung homeostasis [12]. To fulfill their versatile physiological roles, AMs exhibit pronounced heterogeneity and functional plasticity, enabling them to dynamically adapt their cellular functions in response to microenvironmental stimuli [12,13,14,15]. The macrophage activation states are commonly classified into classically (M1) and alternatively activated (M2) phenotypes [16]. M1 macrophages resemble a proinflammatory phenotype that is associated with the release of proinflammatory cytokines, whereas M2 macrophages are mainly involved in inflammation resolution and tissue-repair activities [14,16,17,18]. Dysregulated or imbalanced activation of macrophages can promote harmful effects to the lung, resulting in chronic inflammation, or pathological tissue remodeling [14,17,19,20,21]. Consequently, pulmonary delivery systems must be designed to enable safe interaction with these cells.

AMs are localized within the airway lumen, enabling potential targeting via inhalation. This local route of administration minimizes systemic side effects, enhances local bioavailability and ensures a high degree of patient compliance [22,23,24]. For pulmonary targeting of AMs, the carrier must meet several key requirements: First, the particle needs to have appropriate aerodynamic properties to ensure deposition in the deep lung. Generally, this requires a mass median aerodynamic diameter (MMAD) < 5 µm [25]. Second, the particle needs to be internalizable by the target cell, preferably through exclusive uptake pathways. An elegant strategy is to exploit the intrinsic clearance function of AMs to internalize and digest inhaled particles and microorganisms [12]. AMs are professional phagocytic cells and therefore capable of engulfing particles > 0.5 µm via phagocytosis [26,27]. This uptake mechanism is unavailable to non-phagocytosing cells, making particle size a useful factor to accomplish cellular selectivity. Finally, the carrier must be cytocompatible while avoiding the excessive activation of macrophages in either direction, ensuring that pulmonary homeostasis is maintained. Considering AMs’ pivotal role in particle recognition and clearance following inhalation, safe interaction with them is crucial, also when other cells or compartments within the respiratory tract are intended to be addressed.

Following a previously established Trojan-horse-inspired concept [28,29,30,31], rod-shaped, nanostructured microparticles represent an innovative carrier system for pulmonary applications and potential AM targeting. Since aerodynamic behavior is influenced not only by size and density but also by shape, elongated particles can help to overcome the aerodynamic challenges. These rod-shaped particles can align along the airflow, thereby lowering the aerodynamic drag, which enables a higher particle volume at an equivalent aerodynamic diameter compared with their spherical counterparts [29,32]. Furthermore, the micron-scale size promotes preferential uptake by phagocytic cells such as AMs. The nanostructured morphology provides a large surface area, promoting the loading of therapeutic agents and facilitating their subsequent accessibility to the cell [33]. Microrod-based carriers containing a nanostructured silica (SiO_2_) framework were explored previously, including systems that enabled delivery of nucleic cargos [28,30,31]. More recently, a mesoporous SiO_2_-based system capable of simultaneously delivering a macromolecular payload (siRNA) and an endosomal escape enhancer (chloroquine) was introduced, highlighting the versatility of this type of pulmonary delivery platform [34].

In this context, nanoparticulate calcium phosphate (CaP) as a framework represents a promising alternative that may address relevant limitations of earlier SiO_2_ proof-of-concept microrod platforms: CaP is an endogenous constituent of mammalian teeth and bones, which contributes to its high degree of biocompatibility and biodegradability [35,36]. Furthermore, the material possesses intrinsic pH sensitivity, a useful property for targeted, dissolution-driven disintegration within acidic environments such as phagolysosomes. Overall, pH sensitivity may support intracellular disassembly and subsequent clearance of the material from the body, which is an important aspect for pulmonary carrier design. The reported contribution to the endosomal escape through osmotic effects [37,38], enabling the delivery of various macromolecular drugs, including siRNA [39,40,41] and pDNA [42,43], might be an additional asset to be evaluated in the future.

Accordingly, this study aimed to develop a hierarchically engineered, pH-responsive pulmonary delivery system capable of safe interaction with AMs. The formulation was realized using a template-assisted technique, followed by nanoparticle pre-crosslinking using alginate and subsequent electrochemically driven polymer deposition as part of a Layer-by-Layer (LbL) approach [29,44,45]. The integration of LbL into the fabrication process adds high versatility in polymer choice, enabling the potential for incorporation of diverse polyelectrolytes, including nucleic acids or peptides [46]. To maximize biocompatibility and degradability, the polyanion carboxymethyl chitosan (CMC) [47,48] was combined with the polycation protamine (Prot) [49]. For the morphological characterization of the obtained microrods, scanning electron microscopy (SEM) and the in situ capillary flow imaging tool FlowCam^®^ were used. The pH responsiveness of the carrier was evaluated by comparing disintegration in established body-fluid-simulating buffers such as phagolysosomal simulant fluid (PLSF) [50] and Gamble’s solution, which represents typical lung lining fluid [51]. To assess the suitability of the microrods for pulmonary delivery, their aerodynamic properties were tested using a Next-Generation Impactor (NGI). The cellular interaction with respect to particle uptake was analyzed using SEM and confocal laser scanning microscopy (CLSM), and the cytocompatibility was assessed using MTT and LDH assays, benchmarking CaP microrods against a SiO_2_-based reference system using the murine alveolar macrophage cell line MH-S [52]. To investigate the cellular response to the carrier at the gene expression level, RNA-seq analysis was performed to profile genome-wide transcriptomic changes [53,54,55,56]. Overall, this work establishes physicochemical, aerodynamic, and biological prerequisites of a CaP-based pulmonary microrod platform, thereby providing a foundation for future studies involving therapeutic cargo loading and delivery.

## 2. Materials and Methods

### 2.1. Materials

Calcium phosphate nanoparticles (Cat. No. 693898-5G), protamine sulfate, sodium alginate, L-leucine, sodium citrate, magnesium chloride and potassium chloride were purchased from Sigma Aldrich (Steinheim, Germany). Sodium chloride was purchased from Fisher Scientific (Loughborough, UK). Sodium acetate and sodium hydrogen carbonate were purchased from Grüssing (Filsum, Germany). Calcium chloride and potassium phthalate were purchased from Carl Roth (Karlsruhe, Germany). Disodium hydrogen phosphate was purchased from Merck (Darmstadt, Germany). Amorphous silicon dioxide nanoparticles (Silocym^TM^, 200 nm) were purchased from NanoCym (Scottsdale, AZ, USA). Carboxymethyl chitosan was obtained from Heppe Medical Chitosan GmbH (Halle (Saale), Germany). NHS-rhodamine was purchased from Thermo Fisher Scientific (Waltham, MA, USA) and coupled to protamine according to the manufacturer’s protocol, except that the reaction time was extended from 1 h to 24 h. Unreacted dye was removed by dialysis using a 1 kDa molecular weight cut-off membrane (Spectra/Por^®^, Carl Roth, Karlsruhe, Germany). Tetrahydrofuran (THF) was obtained from Thermo Fisher Scientific (Waltham, MA, USA). As template membranes, ipPore^TM^ track-etched polycarbonate membranes with a pore density of 1 × 10^6^/cm^2^ with a nominal thickness of 12 µm and pore size of 3 µm were purchased from 4it4ip S.A (Louvain-la-Neuve, Belgium). Ultrapure water was obtained by using a Sartorius Arium Pro system (Sartorius, Göttingen, Germany).

### 2.2. Cell Culture

RPMI-1640, Dulbecco’s phosphate-buffered saline (DPBS), Hanks’ balanced salt solution (HBSS), dimethyl sulfoxide (DMSO), Triton X-100, 3-(4,5-dimethylthiazol-2-yl)-2,5-diphenyltetrazolium bromide (MTT), lactate dehydrogenase (LDH) Cytotoxicity Detection Kit (Roche, Basel, Switzerland; Cat. No. 11644793001), and 4′,6-diamidino-2-phenylindole solution (DAPI) were obtained from Sigma Aldrich Life Science GmbH (Seelze, Germany). Fetal calf serum (FCS) (Cat. No. F2442-500ML) and Phalloidin Alexa Fluor 488^TM^ were purchased from Thermo Fisher Scientific (Waltham, MA, USA). Ibi-Treat^®^ 8-well plates for CLSM imaging were obtained from Ibidi^®^ GmbH (Gräfelfing, Germany), and Cellstar^®^ 96-well plates were bought from Greiner Bio-One GmbH (Frickenhausen, Germany). For M1 polarization, lipopolysaccharide (LPS) from *Escherichia coli* O55:B5 (Cat. No. L6529-1MG) was purchased from Sigma Aldrich (Steinheim, Germany), and interferon gamma (IFN-γ) (Cat. No. 130-105-778) was purchased from Miltenyi Biotec GmbH (Bergisch Gladbach, Germany). Interleukin 4 (IL-4) (Cat. No. 214-14-5UG) for M2 polarization was bought from Thermo Fisher Scientific (Waltham, MA, USA).

SV-40-transformed murine alveolar macrophages (MH-S) (Cat. No. 300487) were purchased from Cytion (Eppelheim, Germany) and cultured in RPMI-1640 supplemented with 10% FCS in a humidified atmosphere of 5% carbon dioxide at 37 °C. Cells were subcultured upon reaching 70−80% confluence. For experiments, cells at passages between 5 and 14 were used.

### 2.3. Microrod Fabrication

**Figure 1:**
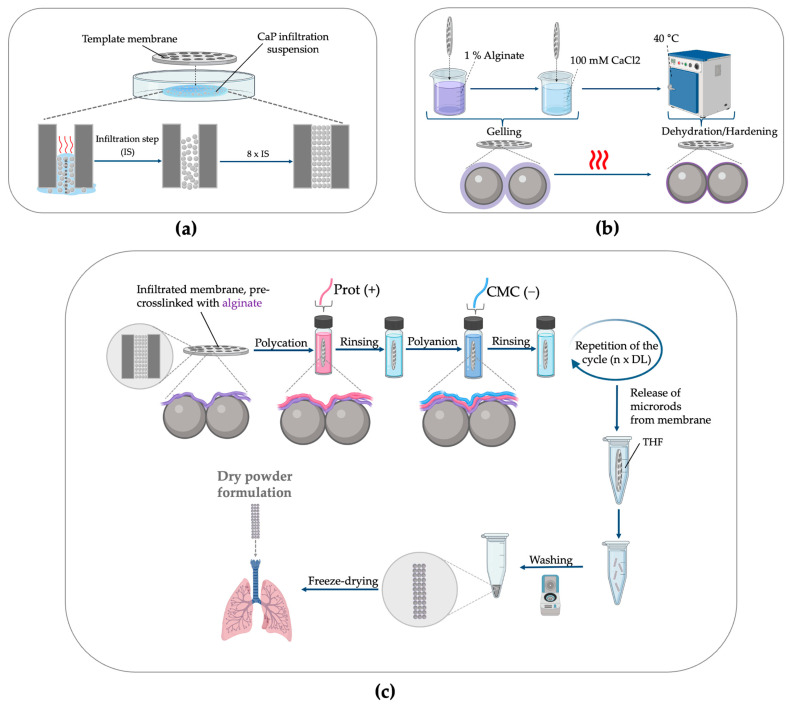
Overview of the microrod fabrication process. (**a**) Infiltration of template membranes with nanosuspension (calcium phosphate (CaP) or silica (SiO_2_) nanoparticles). (**b**) Optional pre-crosslinking of nanoparticles with alginate (Alg). (**c**) Layer-by-Layer crosslinking including the polycation protamine (Prot) and the polyanion carboxymethyl chitosan (CMC) and subsequent rod harvesting. Created in BioRender (Fries, J. (2026), https://BioRender.com/ichnfug, accessed on 20 March 2026).

Rod-shaped, nanostructured CaP microparticles were fabricated according to adapted protocols, as described previously by our group [28,29,30,31,44,45]. In the first step, the pores of template membranes with a nominal length of 12 µm and a pore diameter of 3 µm were infiltrated with a CaP nanoparticle suspension using capillary forces (Figure 1a). To prepare the CaP suspension, CaP nanoparticles were suspended in 0.01 M citrate by using a probe sonicator (Sonoplus HD, Bandelin, Berlin, Germany). Subsequently, 500 µL of the suspension was pipetted onto a Petri dish, after which the membrane was placed on top, followed by drying in an incubator at 40 °C. Residual nanoparticles on the membrane surface were removed by careful wiping with a wetted precision cloth, while excess sodium citrate within the pores was removed by carefully rinsing the pores with water. To achieve dense packing in the membrane pores, the procedure was repeated 4 times for each membrane side. For the SiO_2_ microrods, SiO_2_ nanoparticles were suspended at a concentration of 4 mg/mL in distilled water and infiltrated as described for the CaP microrods.

After the membrane infiltration step, the nanoparticles within the membrane pores were optionally pre-crosslinked with alginate (Alg) (Figure 1b). For this, the membranes were first incubated in a 1% (m/V) alginate solution for 2 min, followed by incubation in 100 mM CaCl_2_ for 2 min. To interconnect the nanoparticles within the pores, the alginate was then dried in an incubator at 40 °C. After drying, excess gel was cleaned off from the membrane with a pre-wetted precision cloth.

To further crosslink the nanoparticles, an LbL approach was employed (Figure 1c). The membrane was first immersed for 4 min in a 0.8% (m/V) Prot solution containing 0.1 M NaCl at pH 7 to achieve charge-driven polycation deposition onto the negatively charged particle surface. Excess polymer on top of the membrane and within the pores was subsequently cleaned by a rinsing step in ultrapure water for 4 min. Afterwards, negatively charged CMC was deposited onto the positively charged surface by immersing the membrane in a 0.2% (m/V) CMC solution containing 0.1 M NaCl. Finally, a second rinsing step removed excess polymer. All polymer solutions used were sterile-filtered using a 0.2 µm bottle-top filter (Nalgene, Thermo Fisher Scientific, Waltham, MA, USA) for long-term storage. This cycle was repeated to obtain different numbers of double layers (DLs). For cellular uptake experiments and evaluation of the aerodynamic properties, NHS-rhodamine-labeled protamine (Prot-Rho) was used to enable fluorescence-based detection. The hierarchically organized polyelectrolyte adsorption during the LbL assembly forms an electrostatically interconnected polymer network among the nanoparticles, thereby stabilizing the rod-shaped superstructure within the pores.

To harvest the microrods, the template membrane was dissolved in tetrahydrofuran (THF) and washed three times using centrifugation (500× *g*, 4 min). Subsequently, the THF was evaporated; the particles were redispersed in ultrapure water and freeze-dried. To test the aerodynamic properties of the formulation, the particles were further coated by redispersing the particles in a 0.4% (m/m) L-leucine solution, followed by another freeze-drying step. The surface coating of the resulting microrods with hydrophobic L-leucine improves the flowability of the dry powder by reducing hygroscopicity and interparticulate cohesion and thereby enhancing aerodynamic properties [57,58,59]. For experiments involving cells, the whole microrod fabrication process was performed under aseptic conditions.

### 2.4. Particle Characterization

To achieve high-throughput analysis of geometric dimensions and counts (for the linearity of the method, refer to Figure S1) of the fabricated microrods, a FlowCam 8000^®^ (Fluid imaging Technologies, Scarborough, ME, USA) imaging system was used. The system has previously been shown to be suitable for characterization and quantification of microrods by Pioch et al. [29]. The device was equipped with an FOV 80 (80 µm depth and 700 µm width) flow cell, a 10× magnification objective and a 0.5 mL pump. Particles were suspended in ultrapure water and introduced into the system. The particles were then measured at a flow rate of 0.1 mL/min and a frame rate of 19 fps using the auto-imaging mode with an auto-calibrated background. Data analysis and acquisition were conducted using VisualSpreadsheet software version 5.4.7 (Fluid imaging Technologies, Scarborough, ME, USA). The recorded images were processed using a value-based filter derived from a library of microrods with different dimensions, to ensure accurate particle identification and measurement.

Additionally, morphological characterization was conducted using SEM. Particles were suspended in ultrapure water and applied onto silica wafers. After the dispersant was evaporated, the particles were sputtered with a gold layer using a Quorum Q150R ES sputter coater (Quorum Technologies Ltd., East Grinstead, UK) with a thickness of about 10 nm. Imaging was conducted at a voltage of 5 kV and varying magnifications using a Zeiss Evo HD 15 Electron Microscope (Carl Zeiss AG, Jena, Germany) equipped with a lanthanum hexaboride (LaB6) cathode. SEM size analysis was performed using FIJI (ImageJ2-based distribution version 2.16.0/1.54p).

Powder X-ray diffraction (PXRD) patterns of the CaP nanoparticles (Figure S2) were recorded at room temperature on a D8-A25-Advance diffractometer (Bruker AXS, Karlsruhe, Germany) in Bragg–Brentano *θ*-*θ* geometry (goniometer radius, 280 mm) with non-monochromatic Cu K_α1,2_ radiation (*λ*_1_ = 154.0596, *λ*_2_ = 154.4426 pm). A 12 µm Ni foil working as a K_β_ filter and a variable divergence slit were mounted at the primary beam side. A LYNXEYE detector with 192 channels was used at the secondary beam side. Experiments were carried out in a 2*θ* range of 7 to 120° with a step size of 0.013° and a total scan time of 2 h.

DLS and zeta potential measurements of CaP and SiO_2_ nanoparticles were performed using Zetasizer Ultra (Malvern Panalytical, Malvern, UK).

### 2.5. Analysis of Aerodynamic Properties

The aerodynamic properties of the CaP microrods, prepared with alginate pre-crosslinking and 3 DLs of Prot-Rho/CMC (CaP-Alg-Prot/CMC (3DL)), were determined using a Next-Generation Impactor (NGI) (Copley Scientific, Nottingham, UK). The general setup followed the standardized NGI configuration described by Marple et al. [60], which was previously adapted by our group [31,37]. The airflow was controlled by using an M1A flowmeter (Copley Scientific, Nottingham, UK) with a flow rate of 60 L/min [61,62]. To prevent particle bounce, each collection stage of the NGI was coated as recommended by USP <601> with a solution consisting of 6 parts Brij 35, 34 parts ethanol and 60 parts glycerol [63]. For each tested batch, approximately 3 mg of the formulation was filled into a size 3 gelatin capsule. The capsule was briefly vortexed to ensure uniform powder distribution, inserted into a HandiHaler^®^ (Boehringer Ingelheim, Ingelheim, Germany), and subsequently punctured. Aerosolization was accomplished by applying vacuum-generated airflow for 4 s twice, in accordance with the manufacturer’s instructions for the device [64]. All collecting stages, the inlet and the pre-separator were rinsed with defined volumes of ultrapure water and collected for quantification. In addition, the capsule was dissolved in ultrapure water to determine the remaining particle mass in the capsule. The mass of deposited particles in each stage and the remaining mass in the capsule were quantified using a microplate spectrophotometer (TecanReader^®^ infinite M200, Tecan, Männedorf, Switzerland) at λ_ex./em._: 542/575 nm with batch-specific calibration curves (for method linearity, refer to Figure S3). Using the calculated masses, the mass median aerodynamic diameter (MMAD), the geometric standard deviation (GSD) and the fine particle fraction (FPF) were determined as described by Abdelrahim et al. [65] and adapted by our group [29,30].

The theoretical estimated aerodynamic diameter was calculated using the general relation, including the dynamic shape factor (Equation (1)) [66]:

(1) $$d_{a}=d_{ve}\sqrt{\frac{\rho_{p}\;C_{c}\left(d_{ve}\right)}{\rho_{0}\;C_{c}\left(d_{a}\right)}\frac{1}{\chi }}$$

$d_{a}:$ Aerodynamic diameter,
$d_{ve}:$ Volume-equivalent diameter,
$\rho_{p}:$ Particle density,
$\rho_{0}:$ Standard density (1 g/cm^3^),
χ: Dynamic shape factor,
$C_{c}$: Cunningham slip correction factor.

For particles in the lower micrometer and nanoscale range, deviations from continuum flow conditions reduce the accuracy of Stokes’ law prediction of the drag force. Consequently, the Cunningham slip correction factor was determined by using the correlation proposed by Davies [67] (Equation (2)):

(2) $$C_{c}=1+\frac{2\lambda }{d}\left(A_{1}+A_{2}e^{-A_{3}d/\lambda }\right)$$

$C_{c}:$ Cunningham slip correction factor,
λ: Mean free path (0.066 µm at 20 °C [66]),
d: Particle diameter,
A: Experimentally determined coefficients (*A*_1_ = 1.257, *A*_2_ = 0.400, *A*_3_ = 0.55) [67].

The literature-based dynamic shape factor χ was calculated using the empirical model described by Lau et al. [68].

### 2.6. Cellular Uptake

To evaluate the uptake of the formulations by alveolar macrophages, 2 × 10^4^ cells/well in 200 µL medium (RPMI + 10% FCS) were seeded in a µ-Slide 8 Well. After 24 h of incubation, the medium was aspirated and replaced with fresh medium containing CaP microrods (pre-crosslinked with alginate, 3 DLs of Prot-Rho/CMC) at a concentration of 0.1 mg/mL. After incubation for 3 h and 6 h, the medium was removed, and the cells were washed twice with HBSS. Subsequently, the cells were fixed at room temperature for 15 min using 4% paraformaldehyde (PFA).

For CLSM imaging, cells were further permeabilized by incubation with 0.1% Triton X-100 for 15 min at room temperature. After two washing cycles with HBSS, the nuclei were stained for 30 min using 600 nM DAPI at room temperature. Following two more washing steps, F-actin was stained using Phalloidin Alexa Fluor 488^TM^ at a concentration of 165 nM for 30 min at room temperature. The cells were finally washed with HBSS and analyzed using CLSM (LSM710, Carl Zeiss AG, Jena, Germany). Cell structures were detected with lasers operating at a wavelength of 405 nm for cell nuclei, 488 nm for F-actin, and 561 nm for rhodamine-labeled microrods.

For SEM imaging, cells were washed with HBSS, fixed with PFA 4% for 15 min at room temperature and further dehydrated in a graded ethanol series by sequential incubation with 20%, 40%, 60%, 80% and 100% ethanol solutions. Afterward, cells were sputtered with a gold layer and analyzed using SEM.

### 2.7. Particle Disintegration

To assess the pH responsiveness of the CaP microrods in vitro, phagolysosomal simulant fluid (PLSF, pH 4.5) was prepared according to a previously described protocol to mimic pH and the ion composition of phagolysosomes [50]. The antifungal agent alkyl-benzyldimethylammonium chloride was omitted; instead, the buffer was sterile-filtered using a 0.2 µm bottle-top filter (Nalgene, Thermo Fisher Scientific, Waltham, MA, USA). To simulate the ion composition and pH of the interstitial fluid of the deep lung, Gamble’s solution [51] (pH 7.4) was prepared and sterile filtered as well.

Three independent batches of CaP microrods for each tested condition were prepared using alginate pre-crosslinking and three DLs of Prot/CMC (CaP-Alg-Prot/CMC (3DL)). Subsequently, particles were incubated at a concentration of 0.05 mg/mL in the respective buffer. To achieve a homogeneous dispersion of the particles in the buffer, the suspensions were initially sonicated using an ElmaSonic P ultrasonic bath (Elma, Singen, Germany) for 30 s at 40% power and a frequency of 37 kHz in sweep mode. Suspensions were incubated at 37 °C under slight shaking, and after various periods (10, 20, 30, 70, 180 and 360 min), the remaining microrods and clusters of microrods were quantified using the FlowCam 8000^®^. The device has previously been successfully employed to quantitatively monitor microrod disintegration in suspension [29,34]. To deagglomerate formed agglomerates prior to each measurement, the suspensions were briefly sonicated again for 10 s (for formulation stability under shear stress refer to Figure S4). To obtain a reference value for timepoint t = 0, three independently prepared batches were dispersed in ultrapure water and measured under identical settings as the reference starting point. Thus, the relative percentage of disintegrated particles was calculated for each timepoint.

In addition to that, SEM imaging of residual particles was performed after 360 min of incubation time in respective buffers. For this purpose, suspensions were centrifuged at 20,000× *g* for 4 min, washed once with ultrapure water to remove residual salts, and centrifuged again. The pellet was resuspended in ultrapure water and applied onto a silica wafer for SEM imaging, as described previously.

For quantification of released Ca and P in PLSF, CaP-Alg-Prot/CMC (3DL) was incubated in PLSF at a concentration of 0.05 mg/mL under slight shaking at 37 °C. After 10 min, 60 min and 360 min, the suspensions were centrifuged (20,000× *g* for 4 min), and the supernatants were collected for ICP-QQQ (triple quadrupole ICP-MS, Agilent 8900 ICP-QQQ, Agilent Technologies, Santa Barbara, CA, USA) analysis of Ca and P. Particle-free PLSF blanks were analyzed in parallel and subtracted from the respective sample values to correct for the elemental background of the release medium.

### 2.8. Cellular Toxicity

The cytotoxicity and cell viability of MH-S cells exposed to CaP microrods were evaluated and compared with SiO_2_ microrods using MTT and LDH assays [69].

For microrod fabrication, both CaP microrods and SiO_2_ microrods were prepared using alginate pre-crosslinking, followed by 3 DLs of Prot/CMC. For the cell-based assays, 2 × 10^4^ cells/well in 200 µL medium (RPMI-1640 + 10% FCS) were seeded into a 96-well plate. After 24 h, the medium was aspirated, and the cells were treated with the rod formulations for 24 h at different concentrations. As a negative control, untreated cells were used, whereas 2% Triton X-100 in the medium served as a positive control to induce maximal cytotoxicity.

For MTT assays, the supernatant was removed, and the cells were washed twice with 200 µL HBSS. Subsequently, 200 µL of MTT solution (0.5 mg/mL in HBSS) was added, and the cells were incubated for 1 h under careful shaking. Finally, the supernatant was removed, and the formed formazan crystals were dissolved in 100 µL DMSO. After 20 min, the absorbance was measured at 550 nm with a reference wavelength of 680 nm by the TecanReader^®^ (Tecan, Männedorf, Switzerland).

The cell viability was then calculated according to

(3) $$Cell\;Viability\;[\%]=\frac{{OD}_{sample\;}-{OD}_{positive\;control}}{{OD}_{negative\;control\;}-{OD}_{positive\;control}}$$

To evaluate membrane integrity, LDH assays were performed. After 24 h of incubation with controls and samples, 25 µL of the supernatant was mixed with 25 µL of LDH reagent solution. After a 30 min reaction time at room temperature, the absorbance was measured at 490 nm with a reference wavelength of 680 nm.

The cytotoxicity was then determined according to

(4) $$Cytotoxicity\;[\%]=\frac{{OD}_{sample}-{OD}_{negative\;control}}{{OD}_{positive\;control}-{OD}_{negative\;control}}$$

To estimate the number of cells present at the start of particle incubation, 2 × 10^4^ cells were seeded in 200 µL of medium. After 24 h of incubation time, the medium was aspirated and replaced with fresh medium. Cells from three wells were then counted using high-definition images taken by using Spark Cyto (Tecan, Männedorf, Switzerland) with a 10× objective. The number of cells was determined by counting 5 representative areas (0.36 mm^2^) per well.

All assays were performed in at least three independent experiments, and the results are presented as the mean ± standard deviation.

### 2.9. RNA-Seq Analysis

#### 2.9.1. Cell Treatment

For RNA-seq, 2 × 10^4^ cells/well in 200 µL of medium (RPMI + 10% FCS) were seeded into a 96-well plate. After 24 h, the medium was aspirated, and the cells were incubated for 24 h with three independently prepared batches of CaP-Alg-Prot/CMC (3DL) and SiO_2_-Alg-Prot/CMC (3DL), suspended at a concentration of 0.1 mg/mL in 200 µL of medium. Additionally, M1 polarization was induced by 24 h treatment with 1 µg/mL LPS and 20 ng/mL IFN-γ, and M2 polarization by 20 ng/mL IL-4, following established protocols for different macrophage models [70,71,72]. Medium-only incubation served as the M0 control. After 24 h of treatment, the medium was aspirated, and the cells were washed twice with HBSS.

#### 2.9.2. RNA Isolation and Library Preparation

For RNA isolation, a Direct-zol™ RNA Microprep kit (Zymo Research, Irvine, CA, USA; Cat. No. R2060) was used, according to the manufacturer’s instructions. In brief, cells were directly lysed in a 96-well plate using Trizol^®^ (Thermo Fisher Scientific, Waltham, MA, USA). To remove DNA, samples were treated with the provided DNAse I. After washing three times, RNA was eluted. Concentrations were measured with a NanoDrop™ 2000c (Thermo Fisher Scientific, Waltham, MA, USA), and RNA was stored at −80 °C.

Sequencing libraries were created using the Smart-seq2 protocol described by Picelli et al. [73]. In brief, 100 ng of RNA from each sample was used for reverse transcription and preamplification PCR. PCR products were purified using Ampure XP beads (Beckman Coulter, Brea, CA, USA) at a 1:0.8 ratio. Concentrations of cDNA were measured with Qubit™ 4 Fluorometer (Invitrogen by Thermo Fisher Scientific, Waltham, MA, USA) and 4 ng tagmented using a Tn5 transposase (Illumina, San Diego, CA, USA; Cat. No. 20034211). Tagmented DNA was barcoded and PCR-amplified, and the PCR product was purified with Ampure XP beads at a 1:0.9 ratio. Concentration of the libraries was measured with Qubit. Libraries were sequenced on an Aviti sequencer (Element Biosciences, San Diego, CA, USA) with 75 bp paired-end reads.

#### 2.9.3. RNA-Seq Data Processing

The raw sequencing data files were processed using the nf-core/rnaseq pipeline (v3.14.0) [74], using Star–Salmon against the mus musculus genome assembly and gene annotations based on Ensembl v114. The resulting BAM files containing the expression values were imported into RStudio v4.4.2 for further analysis. Differential analysis was performed using DESeq2 v1.44.0 [75]. Genes were deemed differential when they had a Benjamini–Hochberg-adjusted *p*-value < 0.05 and an effect size of |log_2_FC| > 0.5.

RNA-seq data have been uploaded to GEO and are available under the accession number GSE317613.

### 2.10. Statistical Analysis

GraphPad Prism 10.06.1 (GraphPad Software, Boston, MA, USA) was used for all statistical analyses, except for RNA-seq analysis. Ordinary one-way ANOVA was performed for non-grouped data sets, and two-way ANOVA was performed for grouped datasets, followed by Tukey’s post hoc test for multiple comparisons. Differences were considered significant at *p* < 0.05 (0.05 = *, 0.01 = **, 0.001 = ***, 0.0001 = ****). For RNA-seq data, differential analysis was conducted using the DESeq2 package in RStudio, as described in Section 2.9.3.

## 3. Results and Discussion

### 3.1. Particle Characterization

**Figure 2:**
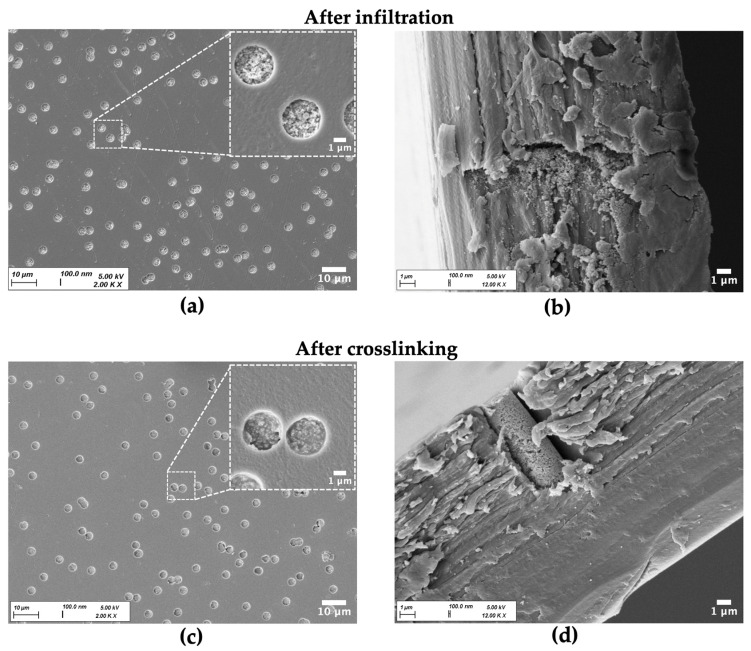
Representative SEM images of template membrane during the fabrication process. (**a**,**b**) After nanosuspension infiltration. (**a**) Top view of membrane with different magnifications showing nanoparticle-loaded pores. (**b**) Pore cross-section illustrating nanoparticle infiltration and absence of structural stabilization. (**c**,**d**) After alginate pre-crosslinking and the first LbL deposition cycle (1 DL). (**c**) Top view of membrane with different magnifications showing formation of the interconnecting polymer network between the nanoparticles. (**d**) Pore cross-section highlighting the stabilized rod-shaped superstructure.

Nanostructured CaP microrods were successfully fabricated by combining a template membrane-assisted technique and subsequent polymer-mediated nanoparticle crosslinking. Using capillary forces for infiltration of the nanosuspension into the template membrane resulted in dense packing of the pores (Figure 2a,b). Alginate pre-crosslinking and subsequent electrostatically driven deposition of the oppositely charged polyelectrolytes Prot and CMC led to the formation of an interconnecting polymer network between the particles (Figure 2b,c).

**Figure 3:**
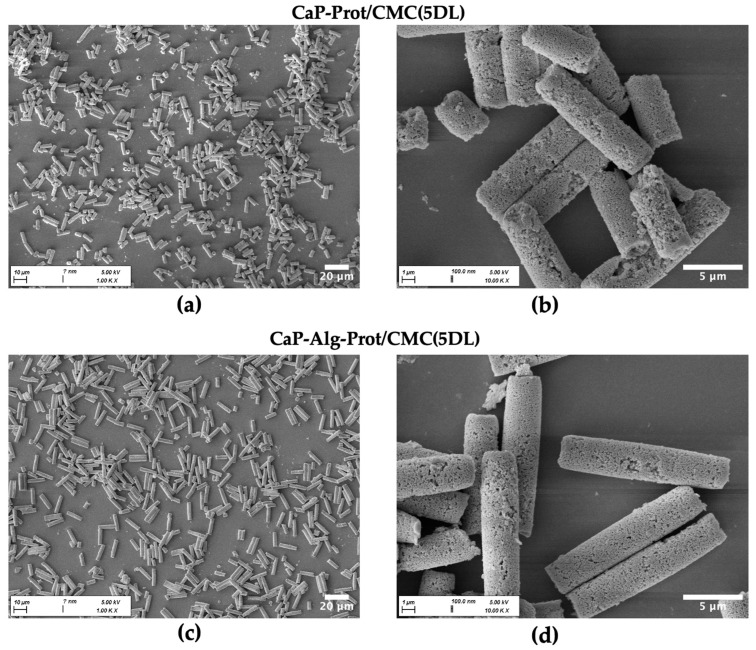
Representative SEM images of CaP microrods with varying magnifications fabricated with and without Alg pre-crosslinking step, followed by five double layers (5 DLs) of Prot/CMC during LbL assembly. (**a**,**b**) Without Alg: higher degree of fragmentation and rougher surface. (**c**,**d**) With Alg: longer microrods with more uniform morphology.

Consequently, the rod-shaped superstructure defined by the membrane geometry was effectively stabilized. SEM close-up images of the yielded microrods after dissolution of the template reveal a cohesive organization of the nanoparticles (Figure 3). Particles fabricated with an alginate pre-crosslinking step exhibit a more uniform shape and higher structural integrity, resulting in fewer fragments compared to those produced without this additional stabilizing step.

**Figure 4:**
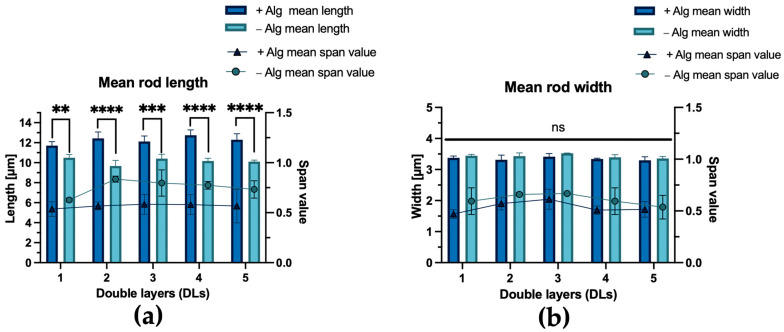
FlowCam^®^ analysis of individual CaP microrods fabricated with (+Alg) and without (−Alg) Alg pre-crosslinking, followed by varying numbers of double layers (DLs) of Prot/CMC. (**a**) Mean length and corresponding mean span value. (**b**) Mean width and corresponding mean span value. Across all tested DL numbers, Alg pre-crosslinking resulted in consistently longer rods and lower corresponding span values, whereas the rod width remained largely unaffected. This indicates a closer agreement of Alg pre-crosslinked rods with the template-defined geometry. Each data point represents the mean ± SD of three independently prepared batches (*N* = 3). Each batch was measured in triplicate (*n* = 3) by FlowCam^®^ (>6000 particles per run were analyzed). (*p* > 0.05 ns, *p* < 0.01 **, *p* < 0.001 ***, *p* < 0.0001 ****).

This observation is further supported by high-throughput image data analysis using FlowCam^®^ (Figure 4). The data show higher mean length values and a narrower length distribution, as reflected by lower span values ((d_90_ − d_10_)/d_50_), for microrods fabricated with an alginate pre-crosslinking step. Across the batches, the mean length obtained with alginate pre-crosslinking ranged from 11.5 µm to 13.3 µm, compared to 9.3 µm and 10.9 µm for particles without this step. Extensive swelling can be ruled out, considering that the width values of the rods do not indicate significant differences between gel-stabilized and gel-free rods (Figure 4b). Moreover, additional DLs during the LbL assembly had no significant influence on the mean rod dimensions. The effect of the alginate pre-crosslinking step on the mean length was further confirmed by SEM-based length analysis (Figure S5), which provides a higher spatial resolution at the expense of throughput compared to FlowCam^®^. Interestingly, when analyzing the length distributions, the majority of the microrods fabricated with alginate exceeded the nominal 12 µm specified by the manufacturer. To clarify this discrepancy, SEM cross-section imaging of three untreated membranes was performed, which revealed an actual membrane thickness of 14.88 ± 0.14 µm for the membrane batch used, explaining the observed length values (Figure S6). This discrepancy did not affect the main conclusions of our study, as the resulting microrods still remained within a size range relevant for pulmonary applications. Moreover, the mean rod length of the particles (approx. 12 µm) was highly consistent with previously reported SiO_2_-based microrods using the same commercially available membrane [29]. This template membrane was selected because its defined pore structure and dimensions were suitable for fabrication of microrods in the targeted size range. In addition, it has been established for preparation of SiO_2_-based microrods in several studies [29,34].

Overall, these results indicate a beneficial effect of the alginate pre-crosslinking step on the structural integrity of the microrods during fabrication. This can likely be attributed to the almost neutral zeta potential of CaP in aqueous dispersions (−3.01 ± 5.03 mV, at neutral pH), which reduces the electrostatic driving force for polyelectrolyte adsorption, making CaP a less suitable substrate for electrostatic-driven polymer deposition [76]. As a result, the total adsorbed mass of polymer is reduced, resulting in a less cohesive polymer network, thereby compromising the structural integrity. Throughout the fabrication process, repeated rinsing and wiping steps, membrane dissolution, and centrifugation steps generate forces that dislodge insufficiently bound nanoparticles. This may also explain why increasing the number of DLs did not translate into longer rods or narrower length distributions. In contrast, the introduction of a dehydrated alginate-based gel network, which has also previously shown effective stabilization in a SiO_2_-based system [29], already ensures interconnection of the nanoparticles prior to LbL assembly. In addition, alginate introduces negatively charged carboxylate groups into the scaffold, thereby improving the electrostatic basis for subsequent polymer deposition during LbL assembly. The subsequent scalable LbL further enables the incorporation of functional polyelectrolytes for potential drug loading. Nevertheless, while pre-crosslinking demonstrated beneficial effects, stabilizing the system exclusively with LbL also represents a conceptually viable approach. This may be particularly attractive for systems that are intended to be fully biodegradable by mammals, where the absence of alginase limits alginate degradation [77]. However, given the superior structural integrity and handling stability of the alginate-containing microrods, all subsequent experiments in this study were performed using microrods pre-crosslinked with alginate and three DLs of Prot/CMC (CaP-Alg-Prot/CMC (3DL)).

**Figure 5:**
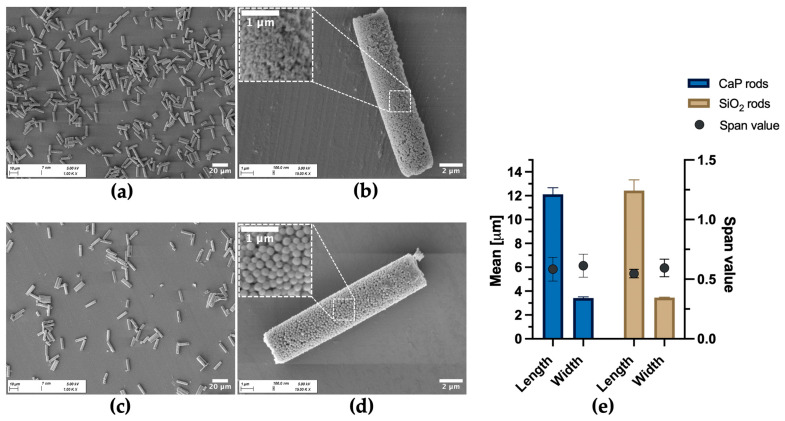
SEM images showing morphological differences between CaP and SiO_2_ microrods and FlowCam^®^ analysis of rod dimensions. (**a**,**b**) CaP microrods at increasing magnifications; (**c**,**d**) SiO_2_ microrods at increasing magnifications; magnified insets highlight differences in nanoparticulate surface, revealing a more uniform nanostructure of the SiO_2_ microrods compared to the CaP microrods. (**e**) Mean length and width, and corresponding span values, determined by FlowCam^®^, indicating consistent dimensions for both microrod types. Each data point represents the mean ± SD of three independently prepared batches (*N* = 3), each measured in triplicate (*n* = 3). In total, >380,000 particles for each type were evaluated. No statistically significant differences were observed for the rod dimensions (*p* > 0.05).

The dimensions of the fabricated SiO_2_ microrods (SiO_2_-Alg-Prot/CMC (3DL)) were highly consistent with those of the CaP microrods (Figure 5), supporting the versatility of the fabrication method. Morphologically, the SiO_2_ microrods exhibited a more ordered architecture, which can be attributed to the uniform spherical shape and narrow size distribution of the SiO_2_ nanoparticles (z-average of 214.02 ± 0.68 nm and a PDI of 0.03 ± 0.02, measured in infiltration suspension) compared to the rather aspherical and less uniform crystalline CaP nanoparticles (z-average of 199.02 ± 12.00 and nm a PDI of 0.17 ± 0.01, measured in infiltration suspension). X-ray powder diffraction analysis of the CaP nanoparticles (Figure S2) revealed a crystallite size of 25 ± 1 nm from the Rietveld refinements. This indicates that the particles in the citrate suspension are likely polycrystalline or form secondary aggregates.

### 3.2. Aerodynamic Properties

For targeting lung tissue, pulmonary delivery offers great advantages over conventional medical treatments, as it combines high local bioavailability with minimized systemic side effects and improved patient compliance [22,23,24]. A central determinant of pulmonary delivery is the aerodynamic behavior of the carrier. Generally, to reach the deep lung, an MMAD between 0.5 µm and 5 µm is required [25]. Elongated particles are characterized by their aspect ratio (AR), defined as the ratio of particle length (l) to width (w). Their shape enables the particle to align with the airflow, thus making the width the predominant determinant influencing the aerodynamic diameter [34,45].

Accordingly, the aerodynamic properties of CaP-Alg-Prot/CMC (3DL) were evaluated using an NGI. The results of the analysis, as presented in Table 1, indicate that the formulation generally meets these requirements. Based on the experimentally determined FlowCam^®^ dimensions (Figure 4), the particles exhibited a mean width of 3.41 µm and a mean length of 12.12 µm (AR ≈ 3.6). Despite their geometric size, the elongated morphology helps to explain why the aerodynamic size remains within the respirable range. However, with an MMAD of 4.76 ± 0.34 µm, the particles remain at the upper edge of the respirable range. Consequently, aerodynamic performance is less favorable for reaching distal lung regions than reported for SiO_2_-based microrod systems of similar dimensions. The MMAD values of these systems were reported as 3.62 ± 0.35 µm in one study [29] and ranged from 3.73 ± 0.14 µm to 7.31 ± 0.91 µm [34] in another study. Hence, the comparatively larger MMAD represents a disadvantage relative to established SiO_2_ microrods. The difference is most likely related to the higher density of the nanoparticulate scaffold: while the density of amorphous SiO_2_ nanoparticles is commonly referenced in the literature as 2.2 g/cm^3^ [78,79], the crystallographic density of the CaP nanoparticles, as calculated from PXRD data, was 3.09 g/cm^3^. A potential strategy to overcome this limitation could be the use of CaP nanoparticles with a hollow internal structure, which could reduce the density of the scaffold [80].

Nevertheless, marketed products with similar aerodynamic properties do exist, such as Arikayce^®^, used for the treatment of non-tuberculous mycobacterial (NTM) lung infections (MMAD of approx. 4.7 µm [81]). AMs represent an important intracellular niche for non-tuberculous mycobacteria and, thus, a relevant therapeutic target in this context [82,83]. This example illustrates that aerosols in this aerodynamic range may still be clinically relevant, while further optimization would likely improve distal lung deposition. The GSD of 1.38 ± 0.01 indicates a comparatively narrow aerodynamic size distribution and is below reported values for SiO_2_ microrods, which ranged from 2.29 ± 0.90 to 1.46 ± 0.11 [29,34]. In general, the narrow size distribution highlights a clear strength of the template-assisted technique used for microrod fabrication, resulting in particles with a uniform geometry (Figure 4). The FPF of 51.82 ± 7.77% demonstrates that a substantial fraction of the particles lies within the respirable size range and is comparable to reported values for SiO_2_ microrods (approx. 15% to 59%) [29,34]. Taken together, the aerodynamic data indicate that the particles are generally capable of reaching the deep lung and support the suitability of the formulation for pulmonary delivery and AM targeting.

To further contextualize the measured MMAD, the theoretical aerodynamic diameter ($d_{a}$) was calculated using the crystallographic density of CaP (3.09 g/cm^3^) and a density correction factor of 0.64 for random close packing of equal spheres [84]. For the particle dimensions, the experimentally determined FlowCam^®^ values of CaP-Alg-Prot/CMC (3DL) were used (Figure 4). The theoretical dynamic shape factor χ was calculated based on the empirically derived model described by Lau et al. [68], which is applicable for elongated particles.

In agreement with previous observations for SiO_2_ microrods reported by Pioch et al. [29], the particles outperformed the predicted values (Table 2). This deviation is likely attributed to the particles’ porosity: while the internal pore structure’s reduction in effective particle density is included in the calculation, potential aerodynamic drag-reducing effects due to increased particle permeability are not [85]. As a result, the achieved MMAD is around ~1 µm lower than the calculated one, which on its own would not meet the 5 µm threshold. The obtained values were further contextualized by comparing them with the calculated $d_{a}$ of hypothetical CaP spheres and non-porous microrods (Table 2). The spherical counterparts were assigned either a diameter equal to the rod width (d =w) or equal to the volume-equivalent diameter ($d\;=d_{ve}$).

As illustrated in Table 2, this model-based comparison demonstrates the beneficial effects of the shape and nanostructure on the aerodynamic performance of the microrods. Despite their substantially larger geometric volume, the formulation achieved an MMAD that is even lower than that of hypothetical spheres with a diameter equal to their width.

### 3.3. Particle Uptake

After inhalation, alveolar macrophages (AMs) play a central role in clearing particles from the deep lung. They belong to a specialized population of cells capable of engulfing particles > 0.5 µm via phagocytosis [27]. Because this uptake mechanism is not accessible to most other pulmonary cells, particle size can serve as an effective design parameter to accomplish targeted drug delivery to AMs. In addition to this, the particle shape can modulate the interaction with macrophages. Increasing the AR of particles represents a double-edged sword: while elongated particles offer a larger contact surface area to interact with the cell, thereby enhancing initial attachment, their rate of internalization is reduced compared with spherical counterparts [86]. Once inside the cell, however, the increased volume and surface area enable the delivery of a substantial payload.

For rod-shaped microparticles intended to deliver therapeutics to AMs, the central challenge is to harness the aerodynamic benefits of elongation while ensuring that the particles remain efficiently internalizable by the cells. The influence of microrod AR on particle internalization in dTHP-1 cells, including particle geometries similar to the ones used in this study, was previously explored [29]. According to this study, microrods with a mean width of approximately 3.43 µm and mean length of 12.65 µm (AR ≈ 3.7) showed a favorable balance between aerodynamic performance and uptake volume. These dimensions are very similar to those of the present CaP microrod system (mean width of 3.41 µm, length of 12.12 µm; AR ≈ 3.6).

**Figure 6:**
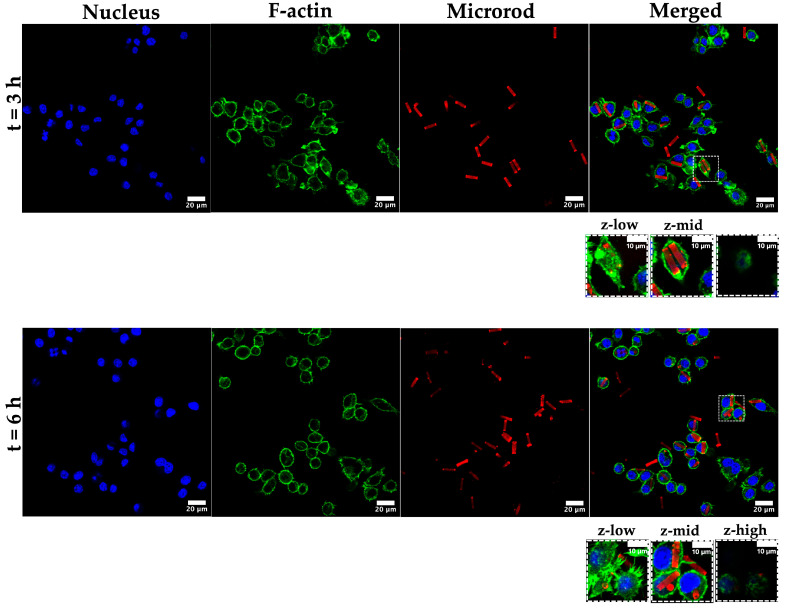
Internalization and interaction of MH-S cells after 3 h and 6 h of incubation time. MH-S cells were incubated with rhodamine-labeled CaP-Alg-Prot/CMC (3DL) for 3 h (top row) and 6 h (bottom row) and imaged by CLSM. Shown are single optical sections (DAPI, blue), F-actin (Phalloidin AF488, green) and microrods (rhodamine, red), as well as merged images. At both timepoints, microrods are successfully internalized by the cells. Image-based evaluation of 8 CLSM images per timepoint revealed that 37.8 ± 7.2% of cells after 3 h and 46.2 ± 14.6% after 6 h were classified as particle-positive (containing at least one internalized microrod or microrod-derived fragment). Boxed sections show different z-planes confirming the localization of the microrods beneath the F-actin-rich cortical region, rather than attached to the cell surface. 3D CLSM reconstructions of the images are shown in Figure S9.

**Figure 7:**
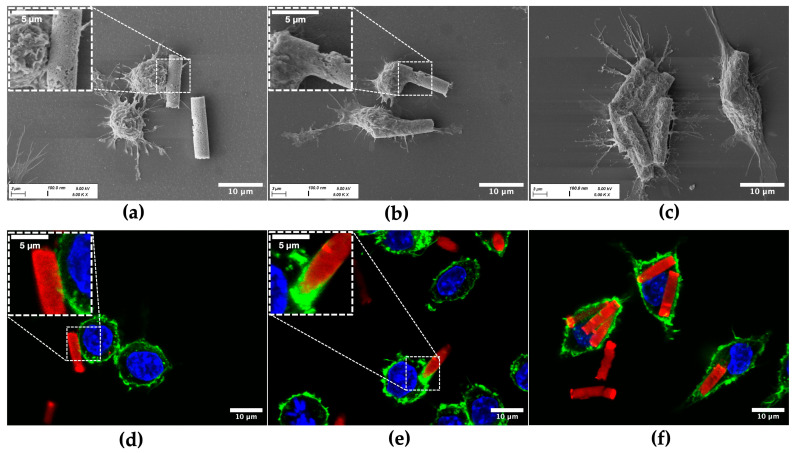
Representative SEM (**a**–**c**) and CLSM (**d**–**f**) images obtained after 3 h of incubation of MH-S alveolar macrophages with CaP microrods, illustrating progressive stages of cell–particle interaction. In CLSM images, nuclei are shown in blue (DAPI), F-actin in green (Phalloidin), and microrods in red (rhodamine). (**a**,**d**) Early contact and particle probing mediated by membrane protrusions; insets provide a magnified view of protrusion–particle contact. (**b**,**e**) Uptake following alignment of the microrods along their shorter axis, consistent with a “tip-first” internalization process; insets highlight F-actin enrichment at rod–cell interface, indicative of phagocytic cup formation. (**c**,**f**) Advanced internalization of microrods.

To investigate potential uptake by alveolar macrophages, MH-S cells were incubated for 3 h and 6 h with rhodamine-labeled CaP-Alg-Prot/CMC (3DL) particles. After fixation, their uptake was analyzed with CLSM and SEM. Nuclei were stained with DAPI, whereas F-actin, a major component of the cytosolic and cortical actin network [87], was stained with Phalloidin AF488. For both timepoints, the successful internalization of a substantial number of microrods was observed (Figure 6). Analysis of different z-planes demonstrated that microrods resided within the cytoplasm, beneath the cortical F-actin, rather than attached to the cell surface. Some macrophages phagocytosed several microrods (Figure 6 and Figure 7c,f), whereas others showed no uptake, reflecting the inherent heterogeneity of AM populations [88]. To complement the qualitative microscopy observations, an image-based evaluation of eight CLSM images per timepoint was conducted. Cells were defined as particle-positive when at least one intracellular microrod or microrod-derived fragment was visible within the cellular boundaries. Across the analyzed fields of view, 37.8 ± 7.2% of cells after 3 h and 46.2 ± 14.6% after 6 h were classified as particle-positive.

Moreover, different stages of cell–particle interaction were imaged (Figure 7). Although the data set consists of static images, the micrographs capture distinct, characteristic stages of phagocytosis [87]. The process typically begins with initial probing of the particle by membrane protrusions (Figure 7a,d). Following the alignment of the rods along their shorter axis, the process proceeds with a “tip-first” engulfment, as reported by Champion et al. [89], consistent with the established shape and orientation dependency of phagocytosis. Accumulation of F-actin at the rod–cell interface (Figure 7e), indicative of phagocytic cup formation [90], enables the cell to wrap its membrane around the shorter axis of the rod. This is particularly relevant for the present microrods (AR ≈ 3.6), since initial contact at the flatter side may promote membrane spreading rather than efficient engulfment, thereby delaying complete internalization [29,89]. Eventually, the internalization process reaches a final stage, in which the particles reside fully intracellularly (Figure 7c,f).

Taken together, these results demonstrate that the microrods remain accessible to macrophage uptake mechanisms despite their elongated geometry and large volume. This fulfils a key prerequisite for AM-targeted pulmonary delivery and provides the cellular entry route necessary for downstream disintegration and potential cargo release.

### 3.4. Particle Disintegration

Over the years, delivery systems that respond to specific stimuli have gained increasing attention. These systems exploit specific microenvironments or external applied stimuli to enable more efficient drug targeting while minimizing undesired off-target effects [91,92]. Especially stimuli inherent to certain microenvironments, such as pH, temperature, ionic strength, or enzymes, can enable trigger-dependent drug release or particle disintegration. Besides this aspect, disintegration of drug delivery systems is also relevant in terms of clearance, preventing toxic accumulation of the material in the body. In this context, the highly active phagolysosomal environment of AMs offers a trigger-rich microenvironment. After phagocytosis, the phagosome undergoes a continuous maturation to eventually fuse with the lysosome, forming the phagolysosome. During this process, the compartment is gradually acidified by the recruitment of v-ATPases, eventually reaching a pH of 4.5 [93]. Therefore, the pronounced pH difference between this intraphagolysosomal compartment and the near-neutral extracellular fluid in the lung offers a promising stimulus to accomplish targeted disintegration of the particles. The CaP matrix of the microrods can therefore serve as the potential pH-responsive element that undergoes acid-driven dissolution within the phagolysosome [38].

**Figure 8:**
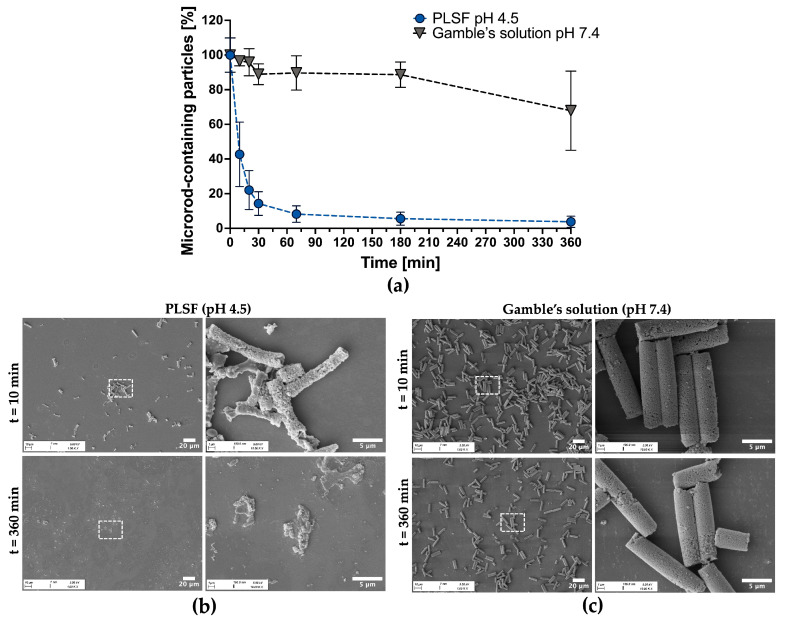
Disintegration of CaP microrods in PLSF (pH 4.5) and Gamble’s solution (pH 7.4). (**a**) Percentage of microrod-containing microparticles relative to the initial timepoint (t = 0), quantified using FlowCam^®^. The t = 0 value was determined from 3 independent batches suspended in water. Data represent mean ± SD of three independent measured batches (*N* = 3). (**b**,**c**) Representative SEM images of remaining microrods after incubation for 10 min and 360 min in PLSF (**b**) and Gamble’s solution (**c**), showing rapid disintegration in acidic conditions and structural integrity at near-neutral pH. Dashed boxes in the overview images indicate the magnified areas displayed to the right.

Accordingly, the pH sensitivity of CaP microrods was tested by comparing their disintegration kinetics in Gamble’s solution, mimicking the pH and ionic strength of extracellular fluid within the lung [51], and in phagolysosomal simulant fluid (PLSF), representing the ion-rich and acidic environment of the late phagolysosome [50]. FlowCam^®^ was used [29,34] to determine the rod concentration at each timepoint, which allowed high-throughput image-based quantification of the remaining microrods and microrod clusters in suspension (for reference images of detected objects, see Figure S7). According to the results (Figure 8a), rapid disintegration at an acidic pH was observed, whereas high structural stability under near-neutral conditions was maintained. Additionally, SEM imaging was performed, which provides a more detailed view of this process (Figure 8b,c).

After 10 min of incubation in PLSF, an already substantial portion of the nanostructured CaP matrix had dissolved (Figure 8b), exposing the underlying polymer network. After 360 min, the CaP phase was almost completely lost, leaving behind residual polymeric complexes. This observation suggests that the porous polymeric layers allow the penetration of protons into deeper regions of the microrod structure. Consequently, dissolution of the CaP matrix is initiated, and the rod superstructure progressively disintegrates. In contrast, the microrods largely retained their structure in Gamble’s solution, while some degree of fragmentation could be observed (Figure 8c). The fragmentation of microrods in an ion-rich environment was previously reported in the literature and can be attributed to charge-screening effects, which compromise the integrity of the polymer network [31,34,94].

Overall, these results demonstrate the pH sensitivity of the formulation and are consistent with dissolution-driven disintegration at an acidic pH, as encountered during phagolysosomal maturation [93]. At the same time, combined with the uptake data (Section 3.2), there is adequate stability under extracellular lung-like conditions to enable uptake by AMs.

In contrast, reported SiO_2_-based reference systems exhibit faster disintegration under conditions simulating the extracellular lung fluid than under acidic conditions resembling phagolysosomes [34]. In these systems, the disintegration behavior is mainly attributed to the destabilization of the polyelectrolyte network, whereas the SiO_2_ framework itself generally lacks rapid degradation or dissolution and may persist in the tissue. In contrast to CaP, the solubility of SiO_2_ is relatively insensitive to pH in the acidic-to-neutral range [95,96], which may also hinder intracellular degradation. Consistent with this, nanoparticulate amorphous SiO_2_ was reported to remain detectable in alveolar and lymph node-associated macrophages even 91 days after exposure in a rat inhalation study [97]. Conversely, the susceptibility of CaP to acidic environments supports intracellular degradation, while the comparatively low solubility at extracellular pH provides sufficient stability for uptake. This is particularly relevant because particles that resist dissolution are predominantly cleared in the distal lung by AMs, whereas particles deposited in the conducting airways are mainly removed by mucociliary clearance [98]. Thus, the CaP matrix provides favorable degradation and resorbability properties in the distal lung region, which may support biocompatibility, reduce long-term persistence and facilitate clearance [99,100]. Nevertheless, direct in vivo studies supporting this process are currently lacking. Beyond this, the acid-triggered dissolution of CaP has been reported to facilitate endosomal escape due to osmotic effects in several studies [39,40,41]. However, this aspect was not investigated in the present work and remains to be demonstrated in future studies.

### 3.5. Cellular Toxicity

**Figure 9:**
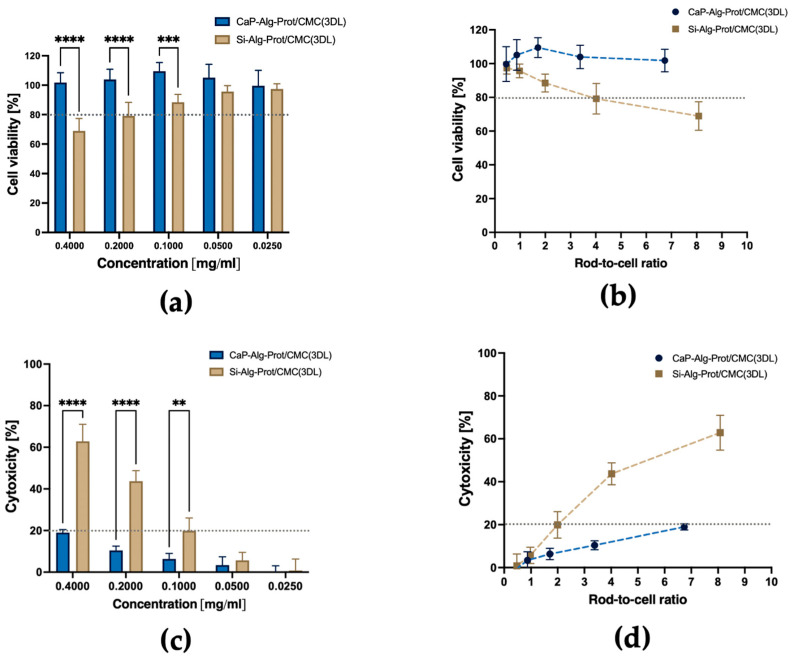
Cytotoxicity and cell viability of MH-S alveolar macrophages after 24 h of exposure time to CaP and SiO_2_ microrods. (**a**) Cell viability assessed by MTT assay at different concentrations [mg/mL]. (**b**) Cell viability expressed as function of calculated rod/cell ratio. (**c**) Cytotoxicity assessed by LDH assay at different concentrations [mg/mL]. (**d**) Cytotoxicity expressed as function of calculated rod/cell ratio. CaP microrods consistently exhibited higher viability and lower cytotoxicity values than SiO_2_ microrods. Each experiment was performed with *n* = 3–6 technical replicates, and data are presented as mean ± SD from *N* ≥ 3 independent experiments. The dotted lines indicate 80% viability and 20% cytotoxicity thresholds for visual reference. (*p* < 0.01 **, *p* < 0.001 ***, *p* < 0.0001 ****) For full statistical analysis refer to Figure S8.

Potential cytotoxic effects of CaP microrods (CaP-Alg-Prot/CMC (3DL)) on MH-S cells were assessed and compared with SiO_2_ microrods (SiO_2_-Alg-Prot/CMC (3DL)) in vitro using MTT and LDH assays [69]. Both rod types were fabricated using the same polymers, to focus specifically on the effect of the nanoparticulate matrix. The assays were performed after 24 h of exposure time with different concentrations of the particles, ranging from 0.025 mg/mL to 0.4 mg/mL, to evaluate possible effects on cell viability and membrane integrity. As illustrated in Figure 9a, CaP microrods did not reduce cell viability across any measured concentration, whereas SiO_2_ microrods caused a concentration-dependent decrease. This trend is further supported by the LDH data (Figure 9c), which indicates a markedly stronger cytotoxic potential of the SiO_2_ microrods. The LDH assay also revealed a concentration-dependent increase in the cytotoxicity of the CaP microrods at higher concentrations. However, all tested CaP microrod concentrations remained below the commonly accepted 20% cytotoxicity threshold [69], while the SiO_2_ microrods exceeded this threshold above 0.1 mg/mL.

Because of density differences between both rod types, identical nominal concentrations (mg/mL) contain different absolute numbers of microrods (Figure S1) interacting with the cells. This aspect becomes particularly relevant since the particles sediment completely during the exposure period. Therefore, the results were additionally normalized to a rod-to-cell ratio (Figure 9b,d).

Accordingly, CaP microrods were well tolerated by the cells at a ratio of ~6.7, indicating a generally favorable safety profile in this alveolar macrophage cell model, permitting exposure to comparatively high particle loads. In contrast, SiO_2_ microrods exceeded the cytotoxicity threshold at ratios above 2. The notably lower cytotoxicity of the CaP microrods may be related to their intraphagolysosomal disintegration. The CaP matrix of particles can dissolve during the phagolysosomal maturation, which may reduce potential mechanical stress on the cell and its membrane. Consequently, membrane integrity seems to be less compromised, which is consistent with the lower release of LDH observed for CaP microrods. In contrast, SiO_2_ microrods were reported to induce significant cytotoxicity towards macrophages in one study [94], while they were well tolerated in others [28,29,30,31]. In addition, another study reported high cytotoxicity of spherical amorphous SiO_2_ microparticles towards MH-S cells specifically, which was associated with endolysosomal leakage [101]. Taken together, these findings suggest that the nanoparticulate CaP framework provides a more favorable cytotoxicity profile and may therefore represent a more suitable candidate for potential clinical translation.

### 3.6. RNA-Seq Analysis

To characterize the transcriptomic response of MH-S cells to CaP microrods, RNA-seq analysis was performed after an exposure time of 24 h. The response was contrasted with that induced by SiO_2_ microrods, serving as a reference for microrods composed of an established nanoparticulate framework [28,29,30,31,34]. As the concentration, 0.1 mg/mL was selected for both materials, as it provided the best balance between sufficient particle exposure and acceptable cytotoxicity, according to the results shown in Figure 9. To better contextualize the cellular response, MH-S cells were also polarized toward M1 using LPS/IFN-γ and M2 using IL-4. Non-stimulated cells served as basal controls (M0). While RNA-seq data were generated from bulk MH-S populations after microrod exposure, they reflect the average transcriptomic response. These population-level signatures provide a mechanistic indication of pathways that are engaged during AM processing of microrods.

**Figure 10:**
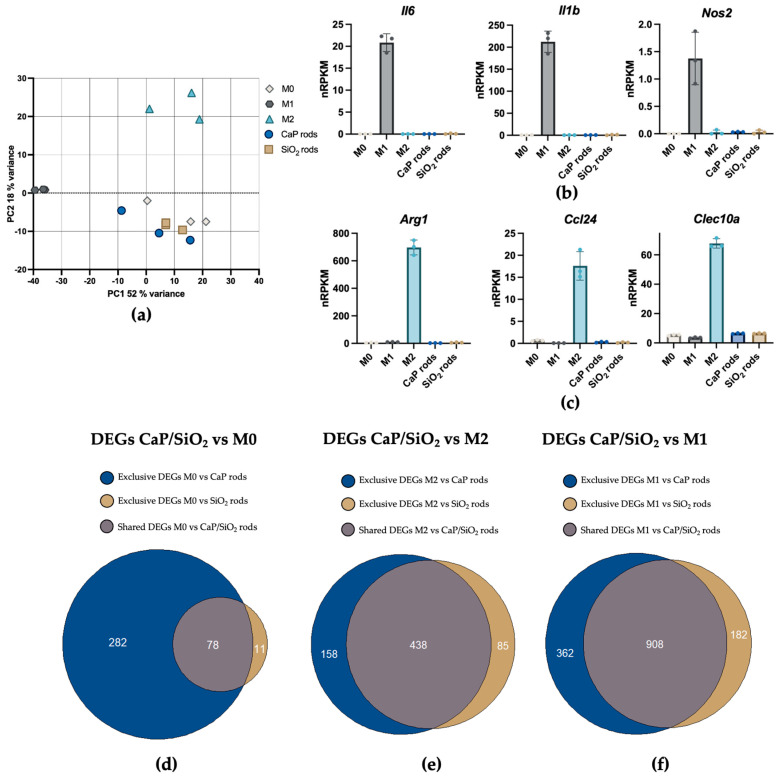
(**a**) Principal component analysis (PCA) of macrophage gene expression profiles after incubation under different conditions: unstimulated (M0), LPS/IFN-γ (M1), IL-4 (M2), CaP microrod exposure and SiO_2_ microrod exposure. PC1 (52%) and PC2 (18%) represent the main sources of transcriptomic variance. Each data point represents one replicate. Both microrod-treated samples cluster near M0, indicating that neither type induces a broad shift towards an M1- or M2-like transcriptomic profile. (**b**) Normalized reads per kilobase million (nRPKs) of representative M1 markers; (**c**) nRPKs of representative M2 markers. (**d**–**f**) Venn diagrams showing overlapping differentially expressed genes (DEGs) of CaP and SiO_2_ microrods compared to M0 (**d**), M1 (**e**) and M2 (**f**). Corresponding heatmaps of DEGs can be found in the Supplementary Materials (Figure S10). Data are based on three independently prepared particle batches (*N* = 3), each tested in a separate well using the same cell passage.

Principal component analysis (PCA) (Figure 10a) of the gene expression profiles revealed a distinct separation of M1- and M2-polarized macrophages and the M0 control, confirming that most of the variance is driven by polarization. In contrast, macrophages exposed to both microrod types clustered closely with the M0 samples, indicating that none of the microrod formulations induced broad transcriptomic changes towards classically or alternatively activated phenotypes. The selective upregulation of established markers for M1 (*Il1b*, *Il6*, *Nos2*) and M2 (*Ccl24*, *Clec10a*, *Arg1*) [102], together with the minimal transcriptomic changes in microrod-exposed cells compared to the basal control, supports this observation (Figure 10b,c). The Venn diagrams illustrated in Figure 10d show that CaP microrod exposure generally resulted in a higher number of differentially expressed genes (DEGs) compared with M0 than the SiO_2_ counterpart, suggesting a broader transcriptomic adjustment to CaP microrods. However, the comparatively small number of identified DEGs for both rod types in comparison to M1 and M2 phenotypes (CaP/SiO_2_ vs. M1/M2) indicates that these transcriptomic changes do not reflect broad macrophage activation but rather small and selective cellular adaptation.

**Figure 11:**
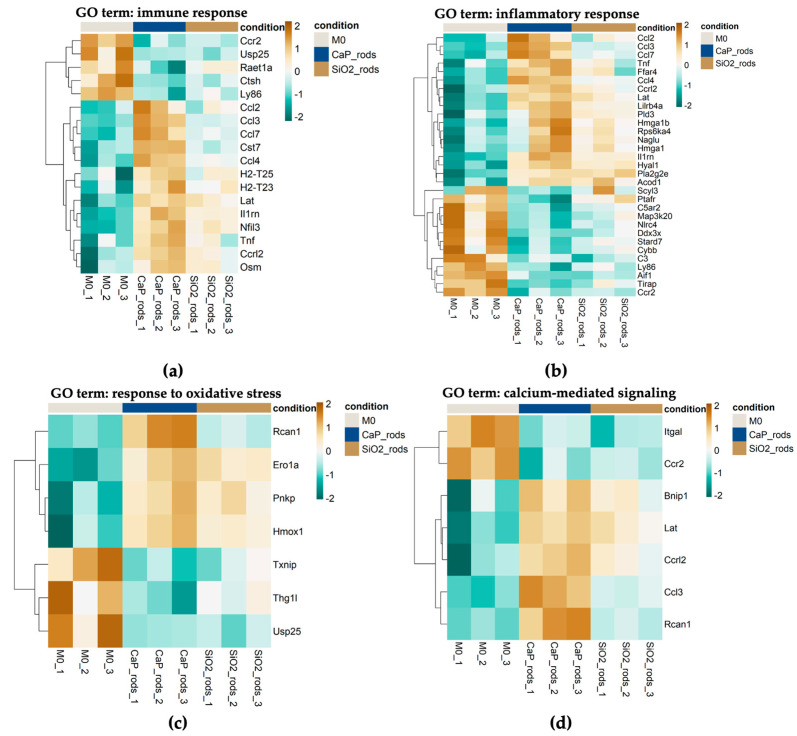
Heatmaps of DEGs in CaP vs. M0 associated with selected Gene Ontology (GO) terms: (**a**) immune response; (**b**) inflammatory response; (**c**) response to oxidative stress; and (**d**) calcium-mediated signaling. Color intensity indicates relative gene expression levels across samples. Overall, compared with exposure to SiO_2_ microrods, exposure to CaP microrods was associated with a more pronounced transcriptomic response across these GO term-related genes. Data are based on three independently prepared particle batches (*N* = 3), each tested in a separate well using the same cell passage.

Given that the M1- and M2-polarized phenotypes primarily served to validate the polarization protocol and to demonstrate that microrod exposure does not induce classical or alternative macrophage activation programs, subsequent computational analyses were restricted to the microrod-treated samples. By excluding strong variance contributed by the polarized reference groups, this recalculated analysis increased the sensitivity and allowed more focused detection of formulation-specific effects. As a consequence, the number of DEGs found in the M0 vs. CaP microrod comparison increased to 543 and that in the M0 vs. SiO_2_ microrod comparison to 99. Gene Ontology (GO) enrichment for the DEGs in the M0 vs. CaP microrod and M0 vs. SiO_2_ microrod comparison (Figure S11) revealed enrichment of immune-related terms such as response to lipopolysaccharide for CaP microrod-exposed cells (Figure S11a) and negative regulation of immune system process for SiO_2_ microrod-exposed cells (Figure S11b). The expression patterns of DEGs associated with the GO terms immune response and inflammatory response (Figure 11a,b) revealed increased expression of several proinflammatory chemokines (e.g., *Ccl2*, *Ccl3*, *Ccl7*) as well as the cytokine gene *Tnf* in the CaP microrod-exposed cells. This indicates that the CaP microrods induced a modestly more pronounced proinflammatory transcriptomic signature in the cells than the SiO_2_ microrods. At the pathway level, upregulation of chemokines such as Ccl2 and Ccl7 suggests a moderate chemokine-associated inflammatory response and may be compatible with a leukocyte recruitment program [103]. Moreover, DEGs related to the GO term response to oxidative stress were also investigated (Figure 11c). The patterns indicate that the oxidative stress response of the cells towards both materials was broadly similar and overall modest, with *Rcan1* representing the most pronounced upregulated gene in CaP microrod-exposed samples. This indicates an overall mild and comparable stress response to both rod types. The expression patterns of DEGs that are included in the GO term calcium-mediated signaling (Figure 11d) indicate that the CaP microrods modulate Ca^2+^-linked signaling pathways, which may be consistent with increased Ca^2+^ availability during intracellular processing and partial dissolution of the CaP matrix. Notably, the magnitude of all changes across the investigated GO terms relative to the M0 control sample was modest overall. Most genes show only moderate deviations, supporting a generally well-tolerated exposure profile at the transcriptome level. Taken together, the cellular response showed minimal macrophage activation, reflecting small-scale adaptations to both materials.

**Figure 12:**
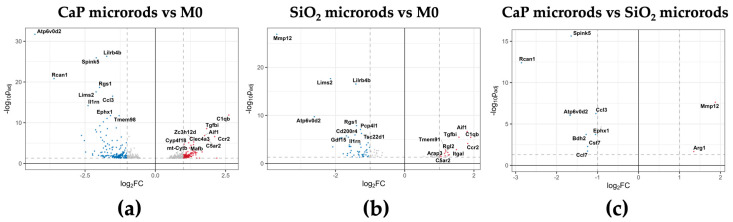
Volcano plots of DEGs: (**a**) CaP vs. M0; (**b**) SiO_2_ vs. M0; and (**c**) CaP vs. SiO_2_. The *x*-axis shows the log_2_ fold change (log_2_FC) and the *y*-axis the −log_10_ adjusted *p*-value (−log_10_p_adj_). Each dot represents a DEG. Only DEGs with an absolute log_2_FC > 1 are highlighted in color. Data are based on three independently prepared particle batches (*N* = 3), each tested in a separate well using the same cell passage.

To further identify individual transcriptomic responses, the most significantly regulated DEGs in each contrast were visualized using volcano plots (Figure 12). In the CaP vs. M0 comparison (Figure 12a), *Rcan1* and *Atp6v0d2* were among the most strongly regulated transcripts. Rcan1 is a feedback inhibitor of the calcium-activated protein phosphatase calcineurin and has been reported to be upregulated by increases in cytosolic Ca^2+^ concentrations. This may suggest the involvement of the calcineurin/NFAT pathway [104,105]. Accordingly, the high upregulation of *Rcan1* may reflect altered intracellular Ca^2+^-linked signaling during CaP microrod processing compared with SiO_2_ microrods (Figure 12a–c). Release of Ca^2+^ ions during partial dissolution of the CaP matrix could potentially increase cytosolic Ca^2+^ ion concentrations and thereby contribute to induction of *Rcan1*. This is supported by ICP-MS-based quantification of released calcium from CaP microrods in PLSF, which demonstrated rapid calcium release under acidic, ion-rich conditions resembling the phagolysosome (Figure S12).

*Atp6v0d2* encodes the d2 subunit of the vacuolar ATPase (V-ATPase) and is involved in phagolysosomal acidification [106]. Although *Atp6v0d2* is also upregulated in SiO_2_ microrod-exposed cells (Figure 12b), the direct CaP vs. SiO_2_ contrast (Figure 12c) indicates a more pronounced induction by CaP microrod exposure. This difference could also relate to the potential dissolution of CaP, which may increase the intraphagolysosomal buffering capacity through the release of phosphate ions. As a result, the phagolysosomal acidification dynamics could differ between both formulations, which in turn may increase V-ATPase-related gene expression. In contrast, in SiO_2_ microrod-exposed cells, *Mmp12* emerged as an important upregulated gene. *Mmp12* encodes matrix metalloproteinase-12 (MMP-12) and is commonly associated with extracellular matrix remodeling. The induction of *Mmp12* following the exposure to amorphous SiO_2_ nanomaterials has also been reported in vivo in rat lung tissue [107]. In general, its overexpression has been implicated in the pathogenesis of several lung diseases including emphysema, COPD and cancer [108]. As a consequence, the upregulation of this gene may damage lung tissue, especially in the context of repeated administration.

Taken together, the transcriptomic response of the cells towards exposure to both rod types was largely coherent, while a subset of DEGs suggests differences in intracellular material processing. The CaP microrods induced more pronounced Ca^2+^-associated transcriptomic changes and showed a tendency toward a moderate inflammatory response, while SiO_2_ microrods triggered a generally less pronounced transcriptomic response. However, the upregulation of *Mmp12* may be relevant in the context of undesired lung tissue remodeling.

## 4. Conclusions

To address the emerging need for advanced and biocompatible drug delivery systems for pulmonary applications, nanostructured CaP microrods were developed. The particles were fabricated by combining two previously described methods for microrod stabilization, a pre-crosslinking step with Alg/CaCl_2_ and subsequent LbL coating, which can, in principle, enable incorporation of macromolecular payloads. In general, this hybrid assembly method may also be applicable to other non-spherical, weakly charged nanoparticles for which purely electrostatic LbL stabilization is less efficient.

The resulting CaP microrods exhibited aerodynamic properties compatible with deep lung deposition, while their elongated geometry still supports uptake by AMs. Consequently, this platform is well suited for pulmonary delivery of LbL-bound therapeutics to the lung and for macrophage-targeted delivery by exploiting the phagocytic uptake pathway.

CaP microrods are a promising alternative to earlier SiO_2_-based systems, preserving the key advantages of the microrod concept. The new system provides advantageous disintegration properties in acidic environments present in phagolysosomes, a biodegradable nanostructured framework and a significantly reduced acute cytotoxicity profile in MH-S cells. In addition, cells that are exposed to this novel type of microrod showed only a moderate proinflammatory response, lacking typical signatures of classical and alternative activation programs. Furthermore, unlike SiO_2_ microrods, CaP microrods did not induce *Mmp12*, which may be favorable for avoiding undesired lung tissue remodeling. Compared with previous proof-of-concept SiO_2_-based systems, the combination of an endogenous and degradable CaP scaffold with biocompatible polymers represents an important step on the way towards clinical translation of the microrod platform.

Taken together, this study establishes CaP microrods as a pulmonary carrier platform and provides key prerequisites for subsequent studies incorporating therapeutic payloads. As a potential drug, oligonucleotides such as pDNA would be attractive cargo, given CaP’s pH sensitivity and the potential for an osmotic contribution to phagolysosomal escape. In addition, the mild proinflammatory response of MH-S cells towards CaP microrod exposure may offer opportunities for application-specific immunomodulation, although this aspect requires dedicated follow-up studies.

## Figures and Tables

**Table 1:** Aerodynamic properties (mass median aerodynamic diameter (MMAD), fine particle fraction (FPF), geometric standard deviation (GSD)) and calculated dynamic shape factor χ of CaP microrods (three tested batches). Individual batch values are provided in Table S1.

MMAD [µm]	FPF [%]	GSD	χ (Calculated)
4.76 ± 0.34	51.82 ± 7.77	1.38 ± 0.01	3.11 ± 0.40

**Table 2:** Calculated theoretical aerodynamic diameters ($d_{a}$) of hypothetical CaP microrods and spheres. Dynamic shape factor χ for microrods was adopted from the literature [68]; χ=1 was assumed for spherical particles.

Particle Type	Internal Structure ^1^	Dimensions	$d_{a}$ (Calculated) [µm]	χ (Literature)
CaP microrod	Porous	w = 3.41 µm, l = 12.12 µm	5.74	2.13
CaP microrod	Non-porous	w = 3.41 µm, l = 12.12 µm	7.19	2.13
CaP sphere (d =w)	Porous	d = 3.41 µm	4.83	1
CaP sphere (d =w)	Non-porous	d = 3.41 µm	6.06	1
$\mathrm{CaP}\;\mathrm{sphere}\;(d\;=d_{ve})$	Porous	d= 5.96 µm	8.41	1
$\mathrm{CaP}\;\mathrm{sphere}\;(d\;=d_{ve})$	Non-porous	d= 5.96 µm	10.53	1

^1^ Porosity was considered only in terms of its effect on density; potential drag-reducing effects of the pore structure were not included in the calculation.

## Data Availability

RNA-seq data have been deposited in GEO under accession number GSE317613. All other datasets are contained within the article and Supplementary Materials or are available upon request.

## Supplementary Materials

The following supporting information can be downloaded at https://www.mdpi.com/article/10.3390/pharmaceutics18040428/s1: Figure S1: Linearity of the microrod quantification method using FlowCam^®^. (a) SiO_2_ microrods (SiO_2_-Alg-Prot/CMC (3DL)). Figure S2: Rietveld refinement of the powder X-ray diffraction data of the CaP nanoparticles. Figure S3: Representative calibration curve demonstrating linearity of the fluorescence measurement for the NGI experiments. Figure S4: Stability of CaP-Alg-Prot/CMC (3DL) microrods under shear stress. Particles were suspended in water and sonicated (37 kHz, 40% amplitude) for 30, 90 and 180 s using an ultrasonic bath. (a) Mean length of the microrods normalized to mean length at 30 s timepoint. (b–d) Length distributions after 30 s (b), 90 s (c) and 210 s (d) of sonication time. Data shown are for three independent batches. Figure S5: Representative length distributions of individual microrods. CaP-Alg-Prot/CMC (5DL) (a,b) and CaP Prot/CMC (5DL) (c,d) determined by SEM (a,c) and high-throughput screening with FlowCam^®^ (b,d). FlowCam^®^ measurements were combined from three independent prepared batches (*N* = 3) (in total, >140,000 particles). SEM measurements were obtained from a single batch (*N* = 1) (220 evaluated microrods). Figure S6: Representative cross-section image analysis of the membranes used, revealing an actual thickness of 14.88 ± 0.14 µm (in total, 3 membranes tested). Table S1: Individual aerodynamic properties (MMAD, FPF, GSD) and calculated dynamic shape factor χ of each tested batch of CaP microrods. Figure S7: Representative images obtained during particle disintegration in PLSF (a) and Gamble’s solution (b) using FlowCam^®^. In PLSF (a), particles disintegrate rapidly, exposing the polymer network. In Gamble’s solution (b), particles remain structurally intact but show a tendency to agglomerate. Figure S8: Full statistical analysis of Figure 9. (a) Cell viability assessed by MTT assay at different concentrations [mg/mL]; (b) cytotoxicity assessed by LDH assay at different concentrations [mg/mL]; (*p* < 0.05 *, *p* < 0.01 **, *p* < 0.001 ***, *p* < 0.0001 ****). Each experiment was performed with *n* = 3–6 technical replicates, and data are presented as mean ± SD from *N* ≥ 3 independent experiments. Figure S9: 3D reconstructions of the CLSM images shown in Figure 6: (a) 3 h incubation and (b) 6 h incubation. Figure S10: DEGs of CaP and SiO_2_ microrods compared with M0 (a,d), M1 (b,e), and M2 (c,f). Data are based on three independently prepared particle batches (*N* = 3), each tested in a separate well using the same cell passage. Figure S11: GO enrichment analysis of (a) CaP microrods vs. M0 and (b) SiO_2_ microrods vs. M0. Figure S12: Calcium and phosphate release from CaP microrods in PLSF (pH 4.5), quantified by ICP-MS. Released amounts of Ca and P were determined after 10 min, 60 min and 360 min and normalized to the initial microrod mass. Values were corrected for the elemental background of the release medium by subtraction of particle-free PLSF blanks. Data are shown as mean ± SD (*N* = 3 independent particle batches).

## Abbreviations

The following abbreviations are used in this manuscript:

AM	Alveolar macrophage
Alg	Alginate
CaP	Calcium phosphate
CLSM	Confocal laser scanning microscopy
CMC	Carboxymethyl chitosan
DEG(s)	Differentially expressed gene(s)
DLs	Double layers
DMSO	Dimethyl sulfoxide
DPBS	Dulbecco’s phosphate-buffered saline
FCS	Fetal calf serum
FPF	Fine particle fraction
GO	Gene Ontology
GSD	Geometric standard deviation
HBSS	Hanks’ balanced salt solution
LbL	Layer-by-Layer
LDH	Lactate dehydrogenase
LPS	Lipopolysaccharide
MH-S	Murine alveolar macrophage cell line MH-S
MMAD	Mass median aerodynamic diameter
MTT	3-(4,5-dimethylthiazol-2-yl)-2,5-diphenyltetrazolium bromide
NGI	Next-Generation Impactor
nRPKMs	Normalized reads per kilobase million
PFA	Paraformaldehyde
PDI	Polydispersity index
PCA	Principal component analysis
PLSF	Phagolysosomal simulant fluid
Prot	Protamine
Prot-Rho	Rhodamine-labeled protamine
PCR	Polymerase chain reaction
PXRD	Powder X-ray diffraction
RNA-seq	RNA sequencing
SEM	Scanning electron microscopy
SiO_2_	Silicon dioxide
THF	Tetrahydrofuran
