# Supplementary material for: pH-Responsive Nanostructured Calcium Phosphate Microrods as Pulmonary Delivery Platform: Fabrication, Characterization, and Comparative Assessment of Cytotoxic and Transcriptomic Responses in Alveolar Macrophages
Source: Pharmaceutics. 2026 Mar 31;18(4):428. doi: 10.3390/pharmaceutics18040428 (PMC13118902; doi:10.3390/pharmaceutics18040428)
Supplement: Supplementary file 1 [file pharmaceutics-18-00428-s001.zip › pharmaceutics-4181670-supplementary.pdf]

Supplementary Materials: pH-Responsive Nanostructured Calcium Phosphate Microrods as Pulmonary Delivery Platform: Fabrication, Characterization, and Comparative Assessment of Cytotoxic and Transcriptomic Responses in Alveolar Macrophages

Jannis Fries, Richard Bachmann, Amalia Schechtel, Oliver Janka, Julia Schulze-Hentrich and Marc Schneider

1. Supplementary information

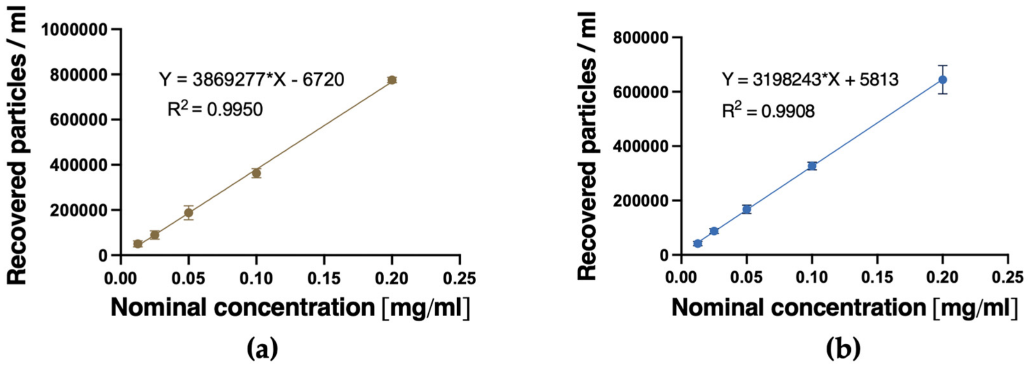

Figure S1. Linearity of FlowCam®-based quantification of individual microrods in suspension. (**a**) SiO_2 microrods (SiO_2-Alg-Prot/CMC(3DL)); (**b**) CaP microrods (CaP-Alg-Prot/CMC(3DL)).

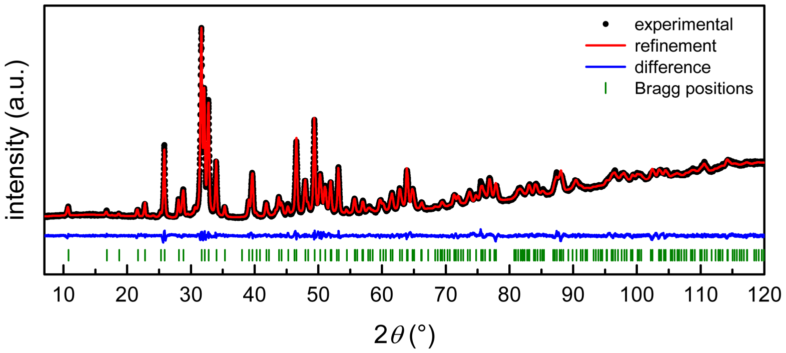

Figure S2. Rietveld refinement of the powder X-ray diffraction data of the CaP nanoparticles.

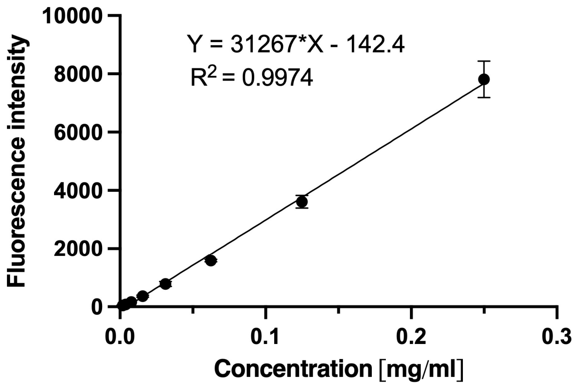

Figure S3. Representative calibration curve demonstrating linearity of the fluorescence measurement for the NGI experiments.

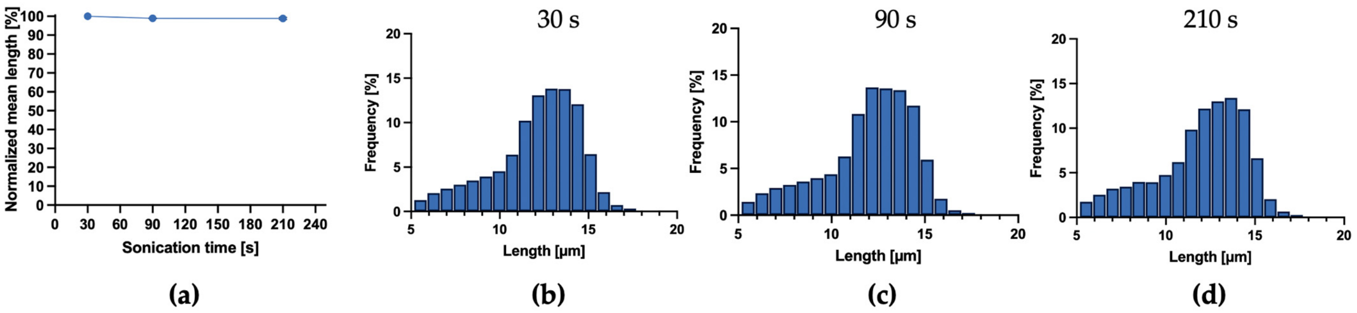

Figure S4. Stability of CaP-Alg-Prot/CMC(3DL) microrods under shear stress. Particles were suspended in water and sonicated (37 kHz 40 % amplitude) for 30, 90 and 180 s using an ultrasonic bath. (**a**) Mean length of the microrods normalized to mean length at timepoint 30 s. (**b-d**) Length distributions after 30 s (**b**), 90 s (**c**) and 210 s (**d**) sonication time. Data shown are for three independently prepared batches.

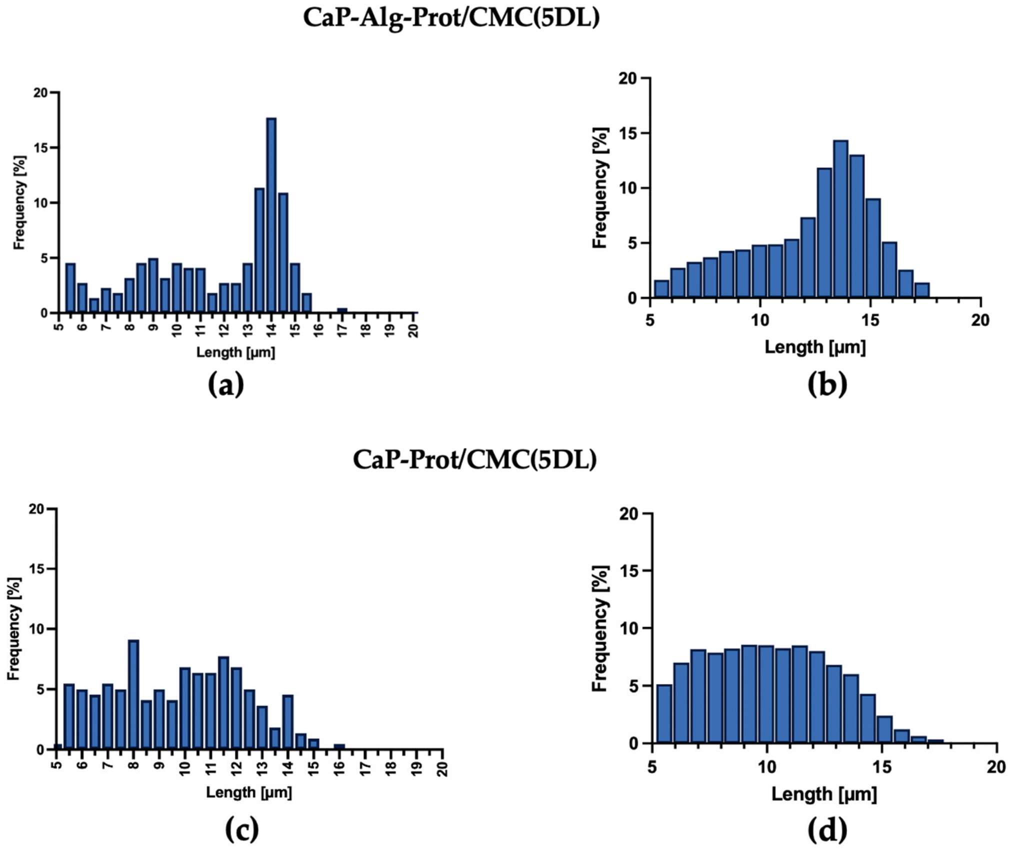

Figure S5. Representative length distributions of individual microrods. CaP-Alg-Prot/CMC(5DL) (**a**,**b**) and CaP Prot/CMC(5DL) (**c**,**d**) determined by SEM (**a**,**c**) and high-throughput screening with FlowCam® (**b**,**d**). FlowCam® measurements were combined from three independent prepared batches ($N = 3$) (in total >140,000 particles). SEM measurements were obtained from a single batch ($N = 1$) (220 evaluated microrods).

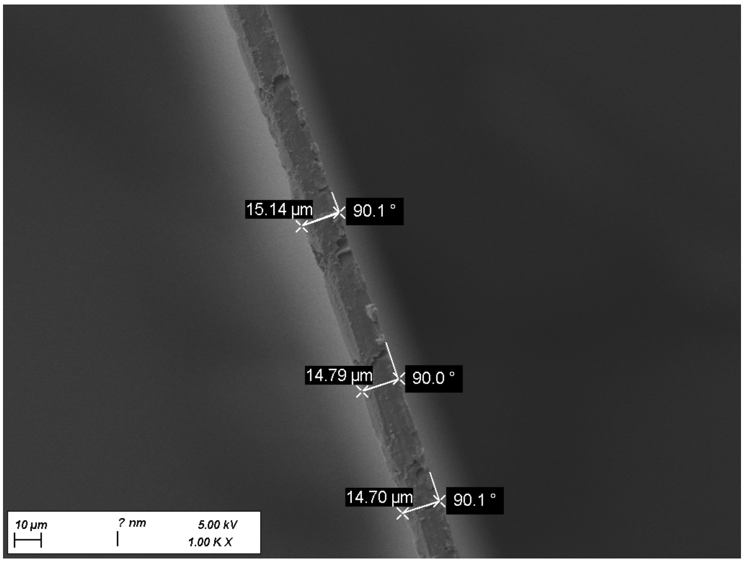

Figure S6. Representative cross-section image analysis of the used membranes revealing an actual thickness of 14.88 ± 0.14 μm (in total 3 membranes tested).

Table S1. Individual aerodynamic properties (MMAD, FPF, GSD) and calculated dynamic shape factor χ of each tested batch of CaP microrods.

Batch	MMAD [μm]	FPF [%]	GSD	χ (Calculated)
Batch 1	4.56	56.72	1.39	3.33
Batch 2	4.49	57.89	1.37	3.45
Batch 3	5.24	40.84	1.38	2.55

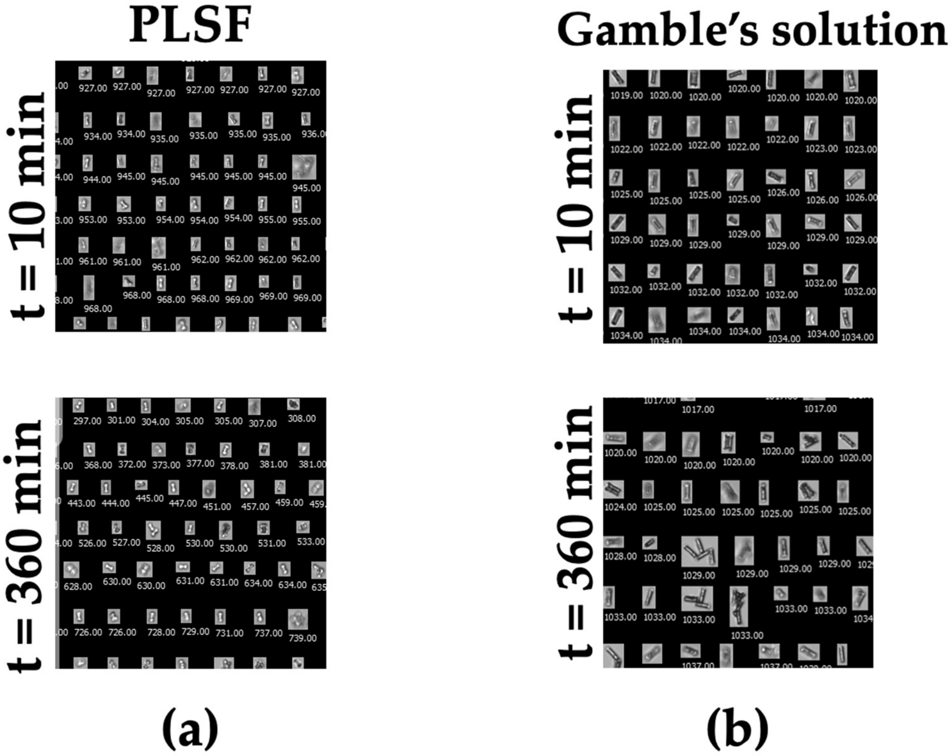

Figure S7. Representative images obtained during particle disintegration in PLSF (**a**) and Gamble's solution (**b**) using FlowCam®. In PLSF (**a**) particles disintegrate rapidly, exposing the polymer network. In Gamble's solution (**b**) particles remain structurally intact, but show a tendency to agglomerate.

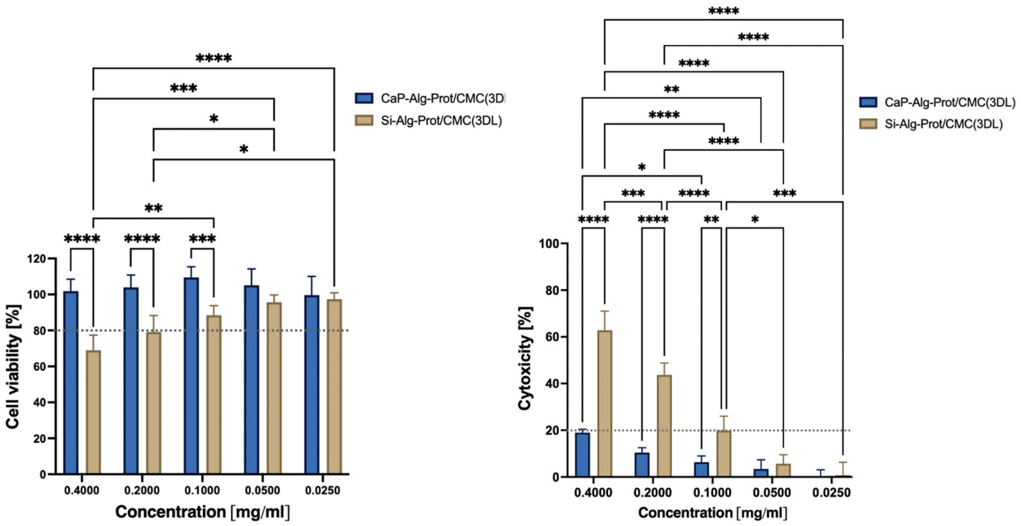

(a) (b)

Figure S8. Full statistical analysis of Figure 9. (**a**) Cell viability assessed by MTT assay at different concentrations [mg/mL]; (**b**) Cytotoxicity assessed by LDH assay at different concentrations [mg/mL]; ($p < 0.05$ *, $p < 0.01$ **, $p < 0.001$ ***, $p < 0.0001$****). Each experiment was performed with $n = 3$–6 technical replicates, and data are presented as mean ± SD from $N \geq 3$ independent experiments.

3 h **6 h**

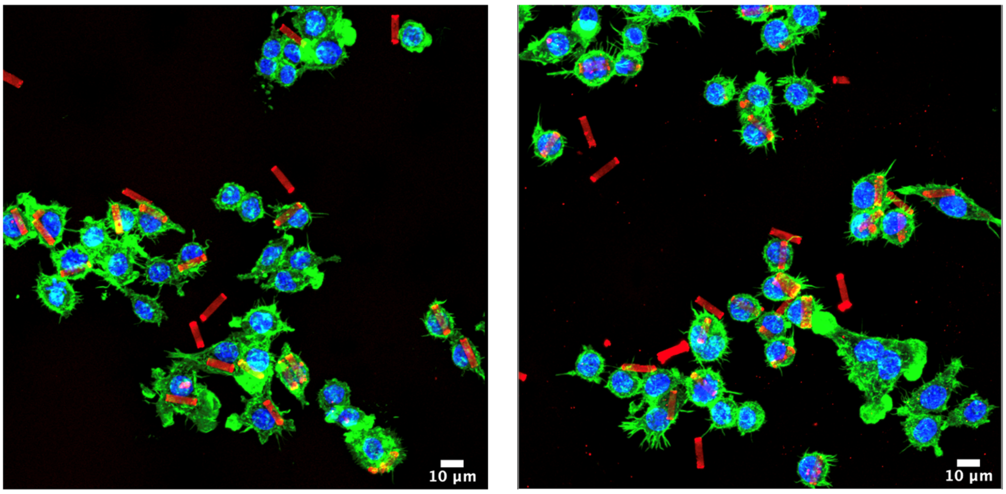

(a) (b)

Figure S9. 3D reconstructions of the CLSM images shown in Figure 6. (**a**) 3 h incubation and (**b**) 6 h incubation.

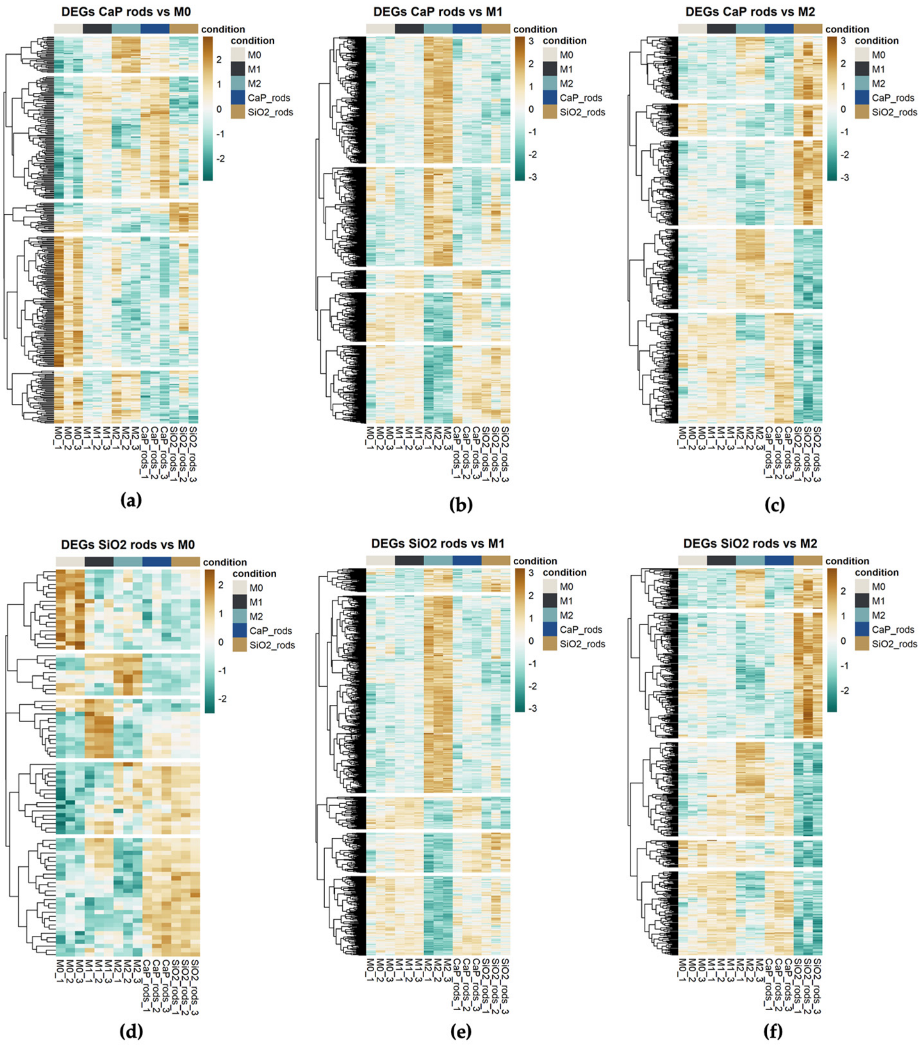

Figure S10. DEGs of CaP and SiO_2 microrods compared with M0 (**a**,**d**); M1 (**b**,**e**) and M2 (**c**,**f**). Data are based on three independently prepared particle batches (*N* = 3), each tested in a separate well using the same cell passage.

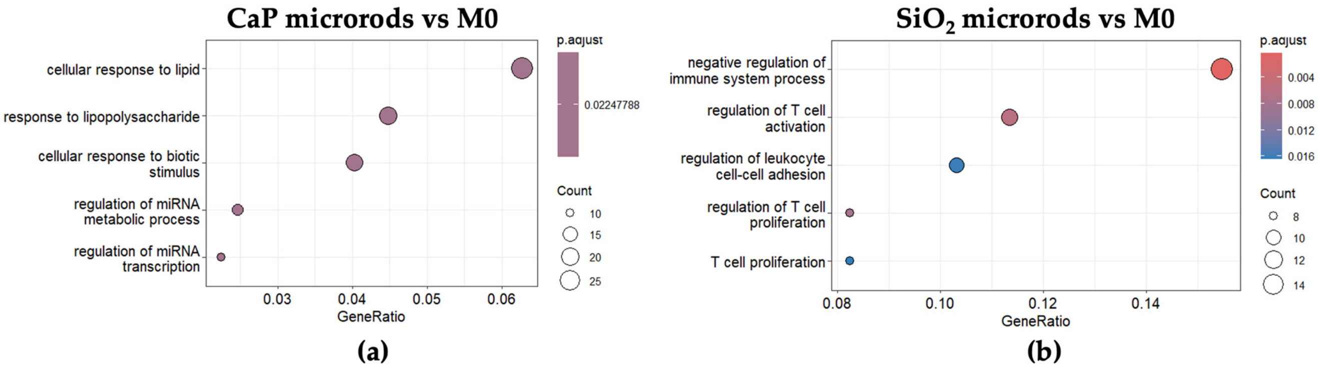

Figure S11. GO enrichment analysis of (**a**) CaP microrods vs M0 and (**b**) SiO_2 microrods vs M0.

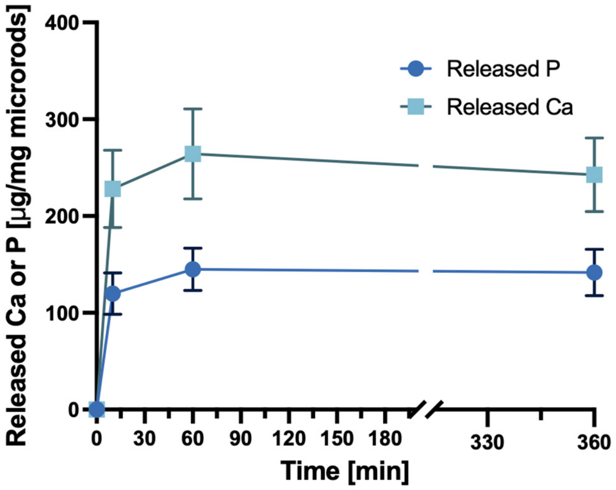

Figure S12. Calcium and phosphate release from CaP microrods in PLSF (pH 4.5), quantified by ICP-MS. Released amounts of Ca and P were determined after 10 min, 60 min and 360 min and normalized to the initial microrod mass. Values were corrected for the elemental background of the release medium by subtraction of particle-free PLSF blanks. Data are shown as mean ± SD (*N* = 3 independent particle batches).
